# Bandgap engineering approach for designing CuO/Mn_3_O_4_/CeO_2_ heterojunction as a novel photocatalyst for AOP-assisted degradation of Malachite green dye

**DOI:** 10.1038/s41598-023-30096-y

**Published:** 2023-02-21

**Authors:** Shaswat Vikram Gupta, Vihangraj Vijaykumar Kulkarni, Md. Ahmaruzzaman

**Affiliations:** 1 Department of Chemistry, National Institute of Technology, Silchar, Assam 788010 India; 2 Department of Civil Engineering, National Institute of Technology, Silchar, Assam 788010 India

**Keywords:** Environmental chemistry, Nanoscale materials

## Abstract

A ternary nanohybrid CuO/Mn_3_O_4_/CeO_2_ was developed in the present work using a co-precipitation-assisted hydrothermal method. The designed photocatalyst's structural, morphology, elemental composition, electronic states of elements, and optical properties were studied using corresponding analytical techniques. Results from PXRD, TEM/HRTEM, XPS, EDAX, and PL showed that the desired nanostructure had formed. Using Tauc's energy band gap plot, it was determined that the nanostructures band gap was ~ 2.44 eV, which showed the band margins of the various moieties, CeO_2_, Mn_3_O_4_, and CuO, had modified. Thus, improved redox conditions led to a substantial decrease in the recombination rate of electron–hole pairs, which was further explained by a PL study in that charge separation plays a key role. Under exposure to visible light irradiation for 60 min, it was revealed that the photocatalyst achieved 98.98% of photodegradation efficiency for malachite green (MG) dye. The process of photodegradation proceeded according to a pseudo-first-order reaction kinetic model with an excellent rate of reaction of 0.07295 min^−1^ with R^2^ = 0.99144. The impacts of different reaction variables, inorganic salts, and water matrices were investigated. This research seeks to create a ternary nanohybrid photocatalyst with high photostability, visible spectrum activity, and reusability up to four cycles.

## Introduction

**Figure 1:**
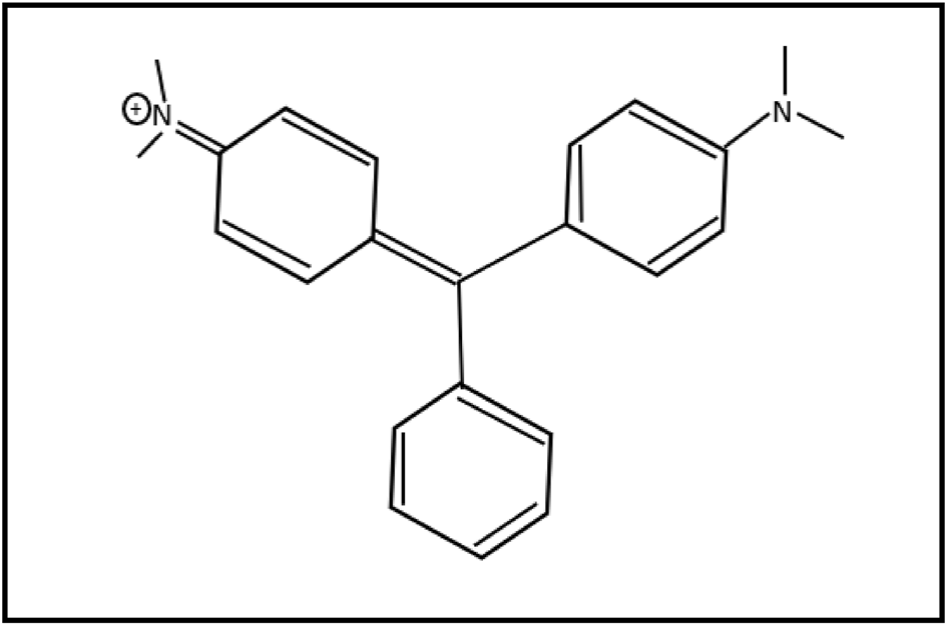
Malachite green (MG) dye chemical structure.

Many professions and industries use organic dyes, including fabric, plastic sheets, leather, medical, skincare, and nourishment, to color their goods and release their effluents into the ecosystem without any primary care^1,2^. These colorful organic pollutants are dangerous to the aquatic environment and human health^3–5^. These coloring agents prevent sunlight from penetrating water streams, slow down the photosynthetic phenomenon in the water ecosystem, and may interact with ionic metals to form chelating complexes, all of which lead to the toxicity of living beings^6^. Malachite green (MG), a cationic dye, is used in many industries as a coloring agent for leather, textile, and woolen goods and in the fishing industry as a parasiticide (Fig. 1). However, MG is known to be a cancer-causing toxin and may seriously harm human health even at minor concentrations (1 mg L^−1^)^7–10^. Initiatives have been taken to keep the water free of these harmful organic dyes like MG dye. In this context, producing an effective nano-sized photocatalyst has garnered much interest^11,12^.

In contrast to other approaches to treating wastewater, like filtration, sedimentation, coagulation, adsorption, etc., photocatalytic degradation entirely breaks down the organic pollutants. It does not produce toxic by-products that have proven resistant to other water treatment approaches^13,14^. Further studies in the field of photocatalysis have resulted in developing a novel short of an oxidative method, which is regarded as the Advanced Oxidation Process (AOP). These techniques aim to completely break down hazardous organic pollutants such as Malachite green, generating reactive oxygen species^15,16^.

The elimination of recalcitrant organic pollutants from water bodies has been accomplished using a kind of AOP called heterogeneous photocatalysis^17,18^. CeO_2_ is a semiconductor with efficient electron mobility, a band gap of ~ 3.27 eV, excellent electrochemical stability, and a good isoelectric point of 9 to catalyst systems^19–21^. Despite multiple benefits, its wide band gap limits its applicability in UV- band region irradiation^22^. Additionally, it has been shown that its function as a photocatalyst is compromised by excessive electron–hole recombination^23,24^. Fine-tuning the band gap of CeO_2_ by combining different semiconductors (metal oxides) with lower band gaps and right band edges would be necessary to maintain a substantial separation of photogenerated electron–hole pairs^25,26^. CeO_2_, in its purest form, has a band gap of about 3.2 eV wide. Pure CeO_2_ nanoparticles cannot efficiently capture visible light because received irradiation lacks the necessary energy to produce charge carriers^27^.

Nevertheless, it has been reported that n–p or n–n heterojunctions having narrow band gap semiconductors such as CeO_2_/CdS^28^, Flower-like CeO_2_/Mn_3_O_4_ microspheres^29^, or CeO_2_/CuO^30^ has significantly improved the separation between the valence and conduction band. Additionally, pairing narrower band gap semiconductors enhances the visible light harvesting potential of the catalyst; as a result, the catalyst's efficiency increases when it is subjected to visible light from the LED. Lately, metal oxides with a ternary composition of materials, including CeO_2_/MgAl_2_O_4_/Mn_3_O_4_^22^, CeO_2_/PAN-ZnO/PAN-Mn_3_O_4_^31^, and CeO_2_/CuO/TiO_2_^32^, have been extensively studied for synergistically boosted photocatalytic efficiency underneath the visible light exposure. Developing a metal oxide ternary heterojunction with a suitable valence-conduction band position can significantly enhance the final nanocomposite's photocatalytic performance. The present study intended to tune the band gap boundaries of CeO_2_ in a way that would be fiscally feasible. Authors of the present study accomplished this by composing CeO_2_ with low-priced nanomaterials such as Mn_3_O_4_ and CuO, each of which has a lower band gap compared to CeO_2_ and band configurations that are suitable for one another. These materials are promising contenders for capturing visible light. The specific band gap of CuO is around 1.79 eV, and coupled systems have shown CuO to have high photocatalytic efficiency. A band gap of 2.27 eV or less in Mn_3_O_4_ might further improve the segregation of photogenerated electrons and holes. Mn_3_O_4_-based photocatalysts have recently shown exceptional responsiveness to visible-light-driven photocatalysis.

In the present work, a simple hydrothermal method for the synthesis of CuO/Mn_3_O_4_/CeO_2_, abbreviated as 'CMCu' as an efficient photocatalyst nanostructure is addressed, and a thorough study is carried out to evaluate the photocatalytic degradation efficiency of the CMCu in the aqueous medium photodegradation of an organic pollutant, Malachite green (MG) dye. For photocatalytic degradation of the MG dye, CMCu, took 60 min, with a photodegradation efficiency of 98.98%. The pseudo-first-order reaction kinetic model with a rate constant of 0.07295 min^−1^ with R^2^ = 0.99144 was observed to be compatible with the photodegradation process. To better understand the application of the designed photocatalyst in the real world, a rudimentary investigation was conducted into the impacts of co-existing substances and real water specimens on the photodegradation of the Malachite green dye (pollutant). This work is innovative because it synthesizes a novel ternary heterojunction of metal oxides, i.e., CMCu, and then uses it to remove a pollutant, MG dye.

## Experimental section

### Reagents and instrumentation for experiment and material analysis

AR-grade reagents were collected from Sigma Aldrich and utilized without further purification. These reagents included cerium nitrate hexahydrate (Ce (NO_3_)_3_.6H_2_O), copper acetate monohydrate (C_4_H_8_CuO_5_), manganese chloride tetrahydrate (MnCl_2_.4H_2_O), sodium hydroxide, Malachite green (C_23_H_25_ClN_2_), and deionized water, etc.

The crystalline nature of CuO/Mn_3_O_4_/CeO_2_ or CMCu and CeO_2_ was evaluated using a Bruker D8 Advance X-ray diffractometer that was irradiated with Cu-K_a_. TEM and SAED studies of CMCu were carried out with the assistance of a JEOL JEM 2100 apparatus. In order to carry out X-ray photoelectron spectroscopy of CMCu, a spectrometer PHI 5000 Versa Prob II was used. Using a JEOL Model JSM—6390LV, an EDAX spectrum and elemental mapping were produced for the CMCu nanocomposite. The Hitachi F4600 instrument was used to obtain data on the photoluminescence of CMCu, Mn_3_O_4_/CeO_2_ (MC), CeO_2_/CuO (CCu), CeO_2_, Mn_3_O_4_, and CuO. The GENESYS 10S UV–visible spectrophotometer was utilized to record the materials' UV–Vis absorbance responses.

### Material fabrication approach

**Figure 2:**
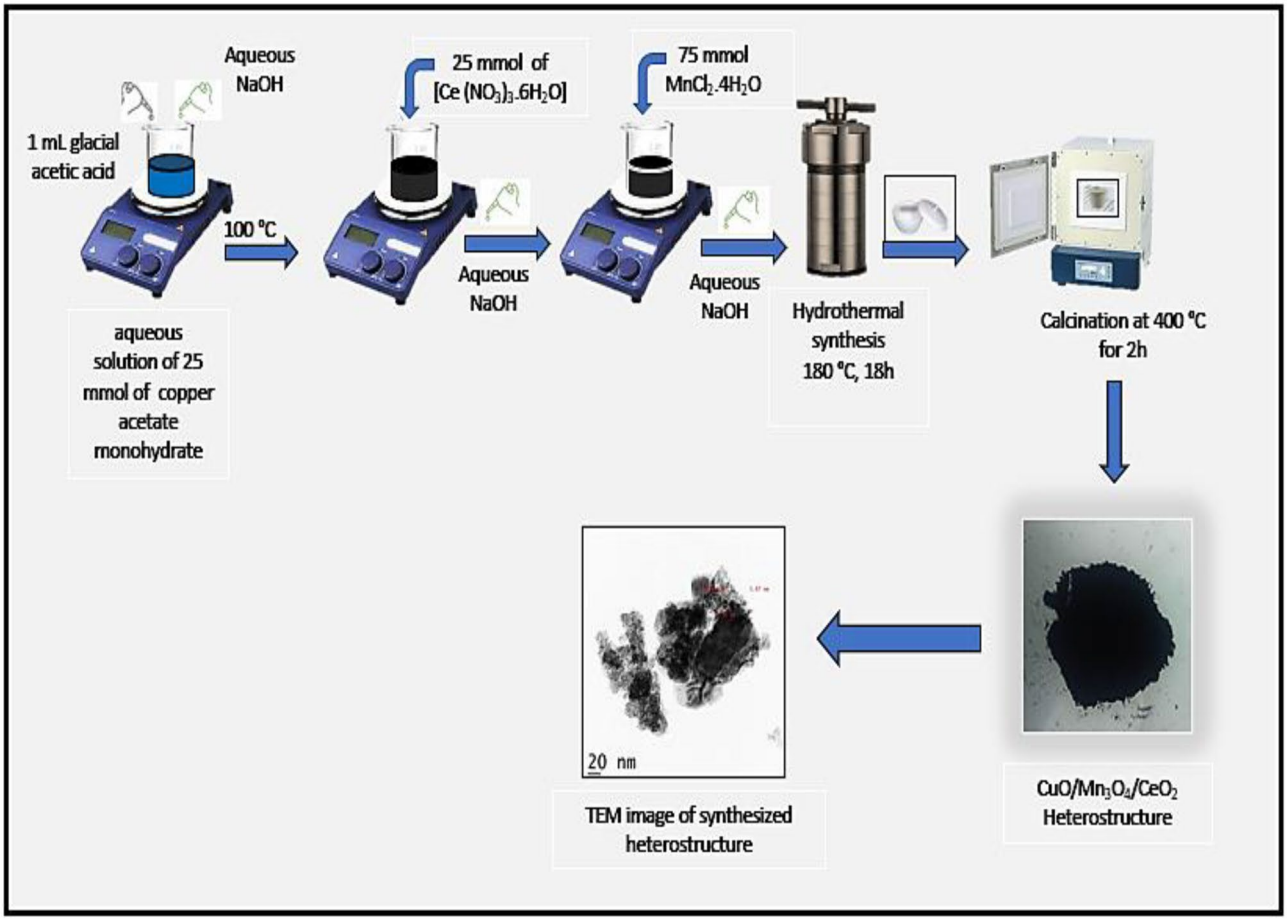
Schematic representation of the synthesis of CuO/CeO_2_/Mn_3_O_4_ heterostructure.

Synthesis of nanomaterials was approached via the one-pot precipitation cum hydrothermal method and followed the previously published work^33^ in the field with some necessary modifications. A glacial acetic acid solution of 1 mL was added to an aqueous solution of 25 mmol of C_4_H_8_CuO_5_ (copper acetate monohydrate) and heated to 100 °C on a hot plate of magnetic stirrer. To this solution drop-by-drop, an aqueous solution of NaOH was added under magnetic agitation. The color of the mixture slowly shifted from blue to black, and a significant quantity of black precipitate of CuO developed. Again, 25 mmol of cerium nitrate hexahydrate (Ce (NO3)3.6H2O) was added to this solution, followed by an injection of dropwise aqueous NaOH solution, and stirred the whole mixture vigorously. Following the addition of 75 mmol of manganese chloride tetrahydrate (MnCl_2_.4H_2_O) and a dropwise injection of an aqueous sodium hydroxide solution to the reaction mixture. The reaction mixture was then transferred to a Teflon autoclave after being thoroughly agitated using a magnetic stirrer. The autoclave was then placed in a 180 °C oven for 18 h. Afterward, a brownish residue was obtained, collected, washed multiple with an ethanol solution, and air dried. A muffle furnace was maintained at 400 °C for 2 h for calcination of an air-dried sample. An identification tag of CuO/Mn_3_O_4_/CeO_2_ (also abbreviated as CMCu) was placed on the calcined sample. The exact process was used to synthesize samples of Mn_3_O_4_/CeO_2_ (MC), CeO_2_/CuO (CCu), CeO_2_, Mn_3_O_4_, and CuO from their respective precursor material. Figure 2 shows the schematic diagram for the synthesis of CuO/CeO_2_/Mn_3_O_4_ heterostructure.

### Experimental setup for efficiency and kinetic appraisal

The photocatalytic efficiency of the developed nanocomposite was determined by observing its capability to disintegrate the MG in its aqueous medium under an LED light. The experiment was conducted in a wooden chamber with a 25 W Philips LED bulb (white light). A lux meter was fixed in an enclosure to estimate light intensity, which was observed at 48.75 W m^2^ at 11,870 lx. The MG dye degradation process was carried out at room temperature. To reach adsorption–desorption equilibrium, the dye solution and photocatalyst were shaken mechanically for 30 min; the absorbance was then measured. The highest absorbance of Malachite green dye at 617 nm was recorded at 10-min increments for 60 min to evaluate photodegradation. The efficiency of degradation was calculated using the given Eq. 1:

1 $$\mathrm{Degradation\,efficiency}\left(\mathrm{\%}\right)=\left(\frac{{\mathrm{C}}_{0}-\mathrm{C}}{{\mathrm{C}}_{0}}\right)\times 100$$

where constants C_0_ and C signify the corresponding MG dye concentrations at t = 0 and t = t, respectively, and Eq. 2 below was employed to evaluate photodegradation kinetics: C_0_ and C denote MG concentrations at t = 0 and t = t, and k is the pseudo-first-order reaction rate constant (in min^−1^).

2 $$ln\frac{{C}_{0}}{C}=kt$$

A reusability test was performed, indicating excellent repeatability until four study repeats under the same parameters. Trapping experiments with different scavengers were performed to clarify the impact of reactive species throughout the photodegradation of MG dye.

## Result and discussion

### Study and evaluation of the structural, morphological, and optical properties of synthesized heterostructure CuO/Mn_3_O_4_/CeO_2_

#### PXRD

**Figure 3:**
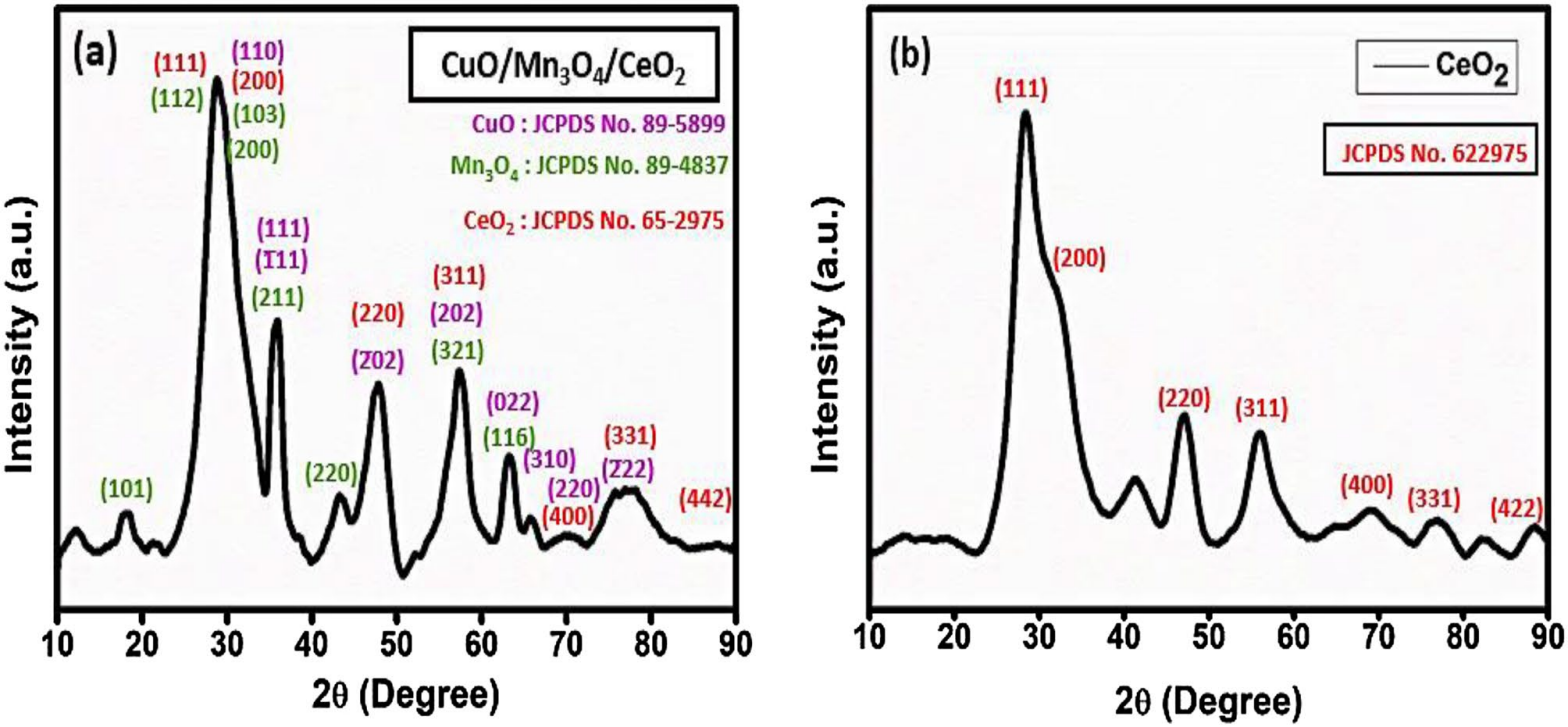
PXRD spectrums of (**a**) CuO/Mn_3_O_4_/CeO_2_ heterostructure and (**b**) pristine CeO_2_.

To identify the crystalline phases of the synthesized heterostructure CMCu, an XRD examination was conducted. The XRD results of synthesized nanocomposite CuO/Mn_3_O_4_/CeO_2_ (CMCu) and pristine CeO_2_ are shown in Figs. 3a, b. The CuO/Mn_3_O_4_/CeO_2_, ternary nanocomposite's XRD data showed peaks of the CeO_2_, Mn_3_O_4_, and CuO phases, labeled in Fig. 3a. In the CeO_2_ phase (JCPDS 65–2975), diffraction from planes (111), (200), (220), (311), (400), (331), and (442) produced peaks at 28.54°, 33.08°, 47.48°, 56.34°, 69.42°, 76.70°, and 88.43°, respectively^34,35^. These peaks matched well with the previously reported JCPDS file number 65–2975 and were compatible with the cubic system with a face-centered lattice structure of CeO_2_ nanomaterial. The cell dimensions of the cubic CeO_2_ with the space group of Fm3̅m [225] are, a = 5.411 Å and α = β = γ = 90°^36^. Peaks were identified at 2Ɵ values of 32.53°, 35.55°, 38.75°, 48.70°, 58.33°, 65.80°, 66.48°, 68.15°, and 75.28°, respectively, correlating to the crystallographic planes (110), (1̅11), (111), (2̅02), (202), (022), (310), (220), and (2̅22) of CuO nanomaterial^37^. These peaks were coherent with the monoclinic system with an end-centered lattice structure of CuO nanomaterial, as well as the results matched with the previously reported literature (JCPDS 89–5899) presenting the cell parameters of the monoclinic CuO with the space group of Cc^9^, are a = 4.689 Å, b = 3.420 Å, c = 5.130 Å, and β = 99.57°^38,39^. Peaks were observed at 2Ɵ values of 18.01°, 28.9°, 31.0°, 32.3°, 36.1°, 44.4°, 58.5°, and 63.2° corresponding to diffraction planes of (101), (112), (200), (103), (211), (220), (321), and (116), respectively and these peaks were consistent with the tetragonal system with body-centered lattice structure of Mn_3_O_4_ nanomaterial, and the data best fit with the earlier reported JCPDS file number 89–4837^40,41^. With the space group of I41/amd [141], the cell parameters of the tetragonal Mn_3_O_4_ are a = 5.763 Å and c = 9. 456 Å^42^. In Fig. 3b, the XRD pattern of the base material of nanocomposite, i.e., pristine CeO_2,_ is given for the best matching of the peaks in the final nanocomposite CuO/Mn_3_O_4_/CeO_2,_ and the peaks well matched with the 2Ɵ values of 28.54°, 33.08°, 47.48°, 56.34°, 69.42°, 76.70°, and 88.43°, and they could be attributed to the CeO_2_ crystalline planes at (111), (200), (220), (311), (400), (331), and (422), respectively, matching with JCPDS 65–2975. JCPDS 65–2975 suggested the face-centered cubic lattice structure of CeO_2_ nanoparticles with cell parameters a = b = c = 5.411 and α = β = γ = 90°. Adition1ay, the crystallinity of the fabricated material was found to be 91.04%, and the average crystallite size was found to be 5.69 nm.

The Debye- Scherrer's equation (Eq. 3) was used to determine the average crystallite size of the fabricated nanoparticles^43,44^. D is the crystallite size (nm), λ (wavelength of CuK radiation) = 1.54056 A, k (shape factor) = 0.89, β (full width at half maximum of the individual peak), and θ is the Bragg's diffraction angle.

3 $$D = \frac{k\lambda }{{\beta \cos \theta }}$$

### TEM, HRTEM, and SAED analyses of the final nanocomposite CuO/Mn_3_O_4_/CeO_2_

**Figure 4:**
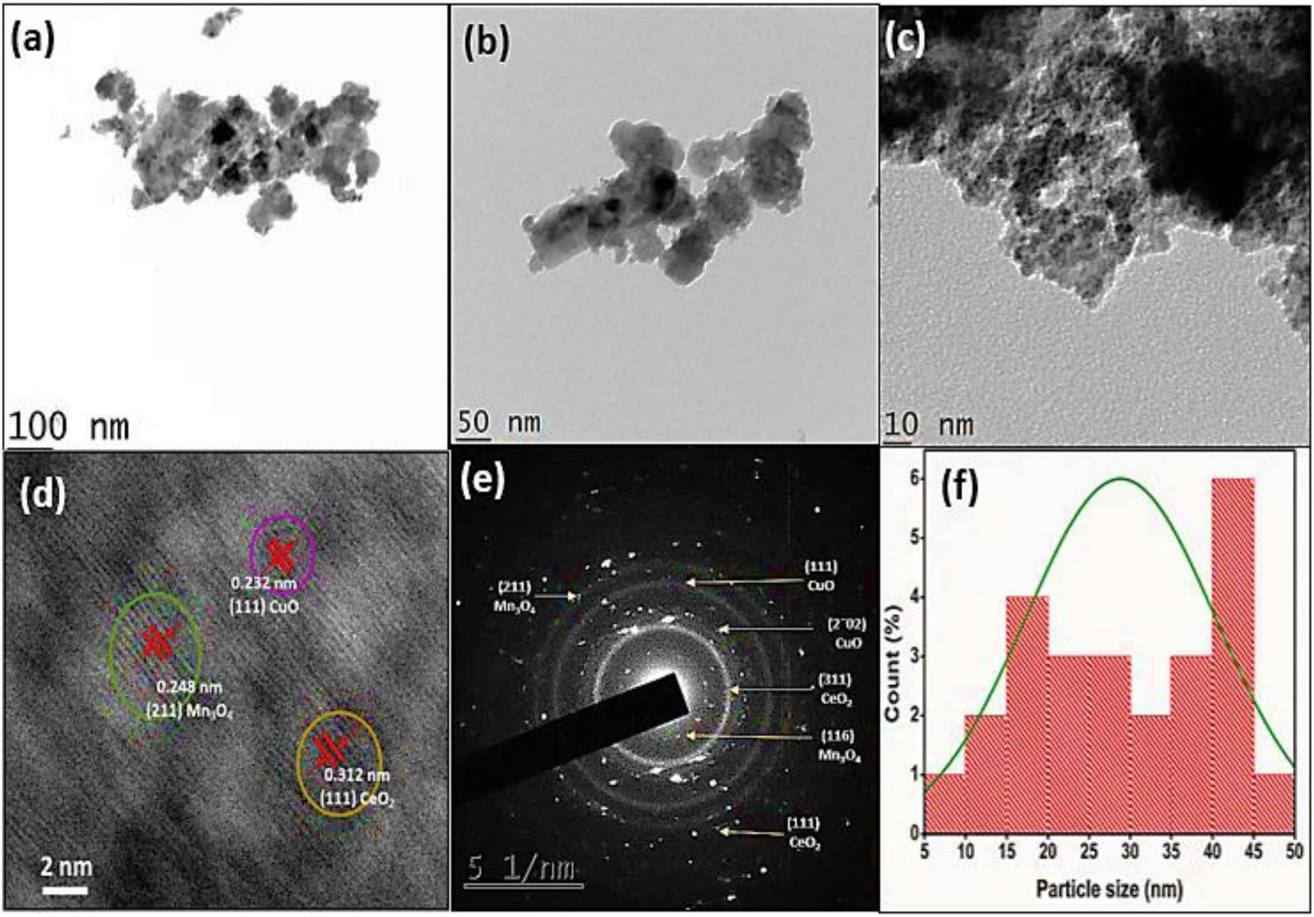
(**a**–**c**) TEM images of CMCu heterostructure at (**a**) 100 nm, (**b**) 50 nm, (**c**)10 nm, (**d**) HRTEM image of CMCu heterostructure at 2 nm, (**e**) SAED pattern of CMCu heterostructure, and (**f**) histogram of CMCu heterostructure for average particle size calculation.

The TEM (Transmission Electron Spectroscopy) method was used so that the morphological features of the developed nanohybrid could be investigated and more information on its structure could be gathered (Fig. 4a–c)). The transmission electron microscopy shown in Fig. 4a displayed images of dispersed nanoparticles of CeO_2_, CuO, and Mn_3_O_4_ in interaction with one another. It is quite likely that inter-facial heterojunctions have indeed been established by these nanoparticles. The nanoparticles were able to accurately identify based on the spacing of the lattice fringes that were visible there in micrographs produced by HRTEM (Fig. 4d). The three distinct lattice fringes had inter-planar spacings of 0.321 nm, 0.248 nm, and 0.232 nm, respectively, for CeO_2_, Mn_3_O_4,_ and CuO nanoparticles. These interplanar spacings might be related to the (111) facet of the CeO_2_ phase (JCPDS 65–2975)^45^, the (211) crystallographic plane of the Mn_3_O_4_ phase (JCPDS 89–4837)^46^, and the (111) facet of the CuO phase (JCPDS 89–5899)^47^. The presence of concentric rings in the SAED patterns (Fig. 4e) was suggestive of the polycrystalline nature of the material^48^. The lattice planes of all three phases were correctly located and marked properly. It was determined that 24.8 nm was the average size of the particles in the final nanocomposite by plotting the histogram (Fig. 4f).

### EDAX and elemental mapping of the final nanocomposite CuO/Mn_3_O_4_/CeO_2_

**Figure 5:**
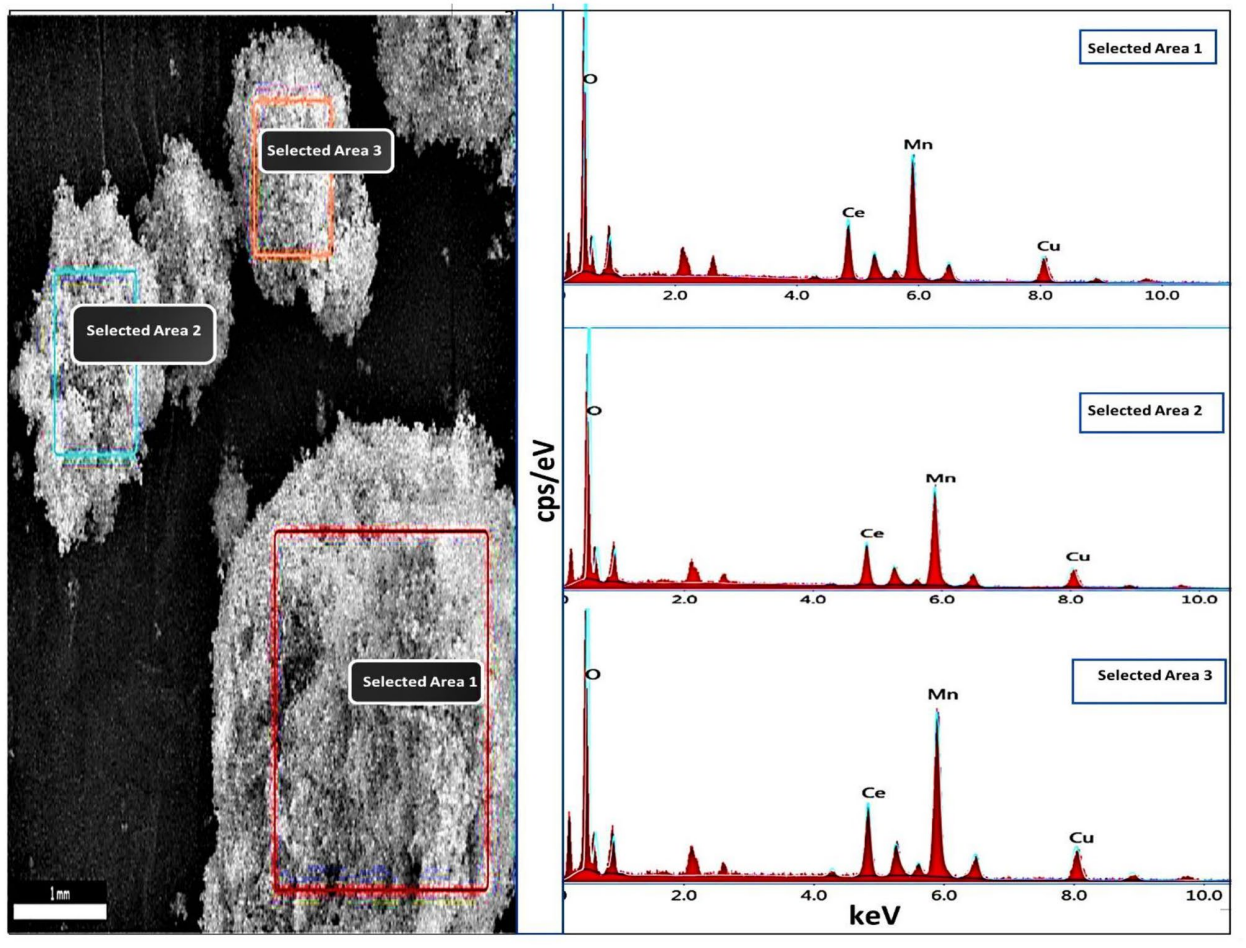
EDAX spectrum of three selected area of CMCu.

**Figure 6:**
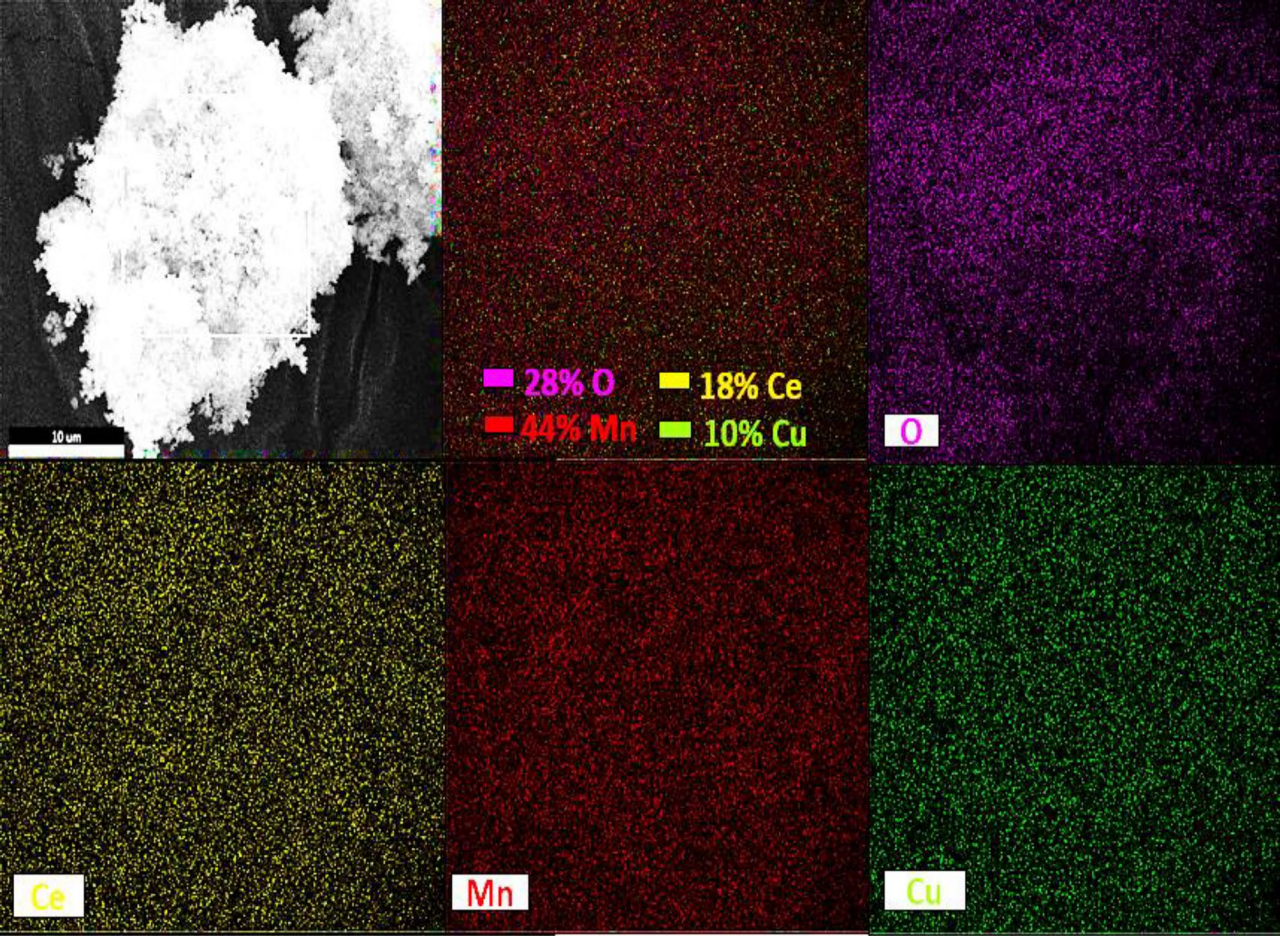
Elemental mapping of CMCu.

The EDAX analysis of three different selected areas (Fig. 5) of the CuO/Mn_3_O_4_/CeO_2_ nanocomposite was performed to gather information about the elements present and their ratios in the synthesized nanocomposite CMCu. EDAX spectrum of all three selected areas exhibited signals that corresponded to Ce, Cu, Mn, and O. The peaks that appear at around 4.8 keV, 8.0 keV, 5.9 keV, and 0.5 keV might be related to Ce, Cu, Mn, and O, respectively, and all of these peaks match to the K-series emissions except Ce peak which matched with L-series emission. Table 1 lists these elements' atomic percentages, implying that a successful synthesis of CeO_2_, CuO, and Mn_3_O_4_ nanomaterials with strong physical coherence among the separate moieties has been placed. In addition, the atomic percentages point to the possible inclusion of CeO_2_, CuO, and Mn_3_O_4_ at the nanocomposites in a ratio of around 1:1:1. Additionally, the absence of contaminant signals (unwanted peaks) in the EDAX spectrum was somewhat compatible with the clear XRD pattern that was produced for the final nanocomposite. The existence of Ce, Cu, Mn, and O in the final nanocomposite was verified yet again through elemental mapping, which can be seen in Fig. 6.

**Table 1:** EDS data of three selected areas of CMCu.

Element	Series type	Selected area								
		**1**			**2**			**3**		
		Weight %	Atomic %	Net Int	Weight %	Atomic %	Net Int	Weight %	Atomic %	Net Int
O	K	26.4	62.0	542.9	29.4	65.1	642.5	23.3	57.8	379.7
Mn	K	33.1	22.7	528.4	33.2	21.4	549.9	35.5	25.6	457.1
Cu	K	13.6	8.1	124.1	13.1	7.3	124.0	14.6	9.1	107.1
Ce	L	27.0	7.3	232.8	24.4	6.2	217.7	26.5	7.5	184.2
Total		100	100		100	100		100	100

### Comparative optical property studies of the single, binary and final nanocomposite

**Figure 7:**
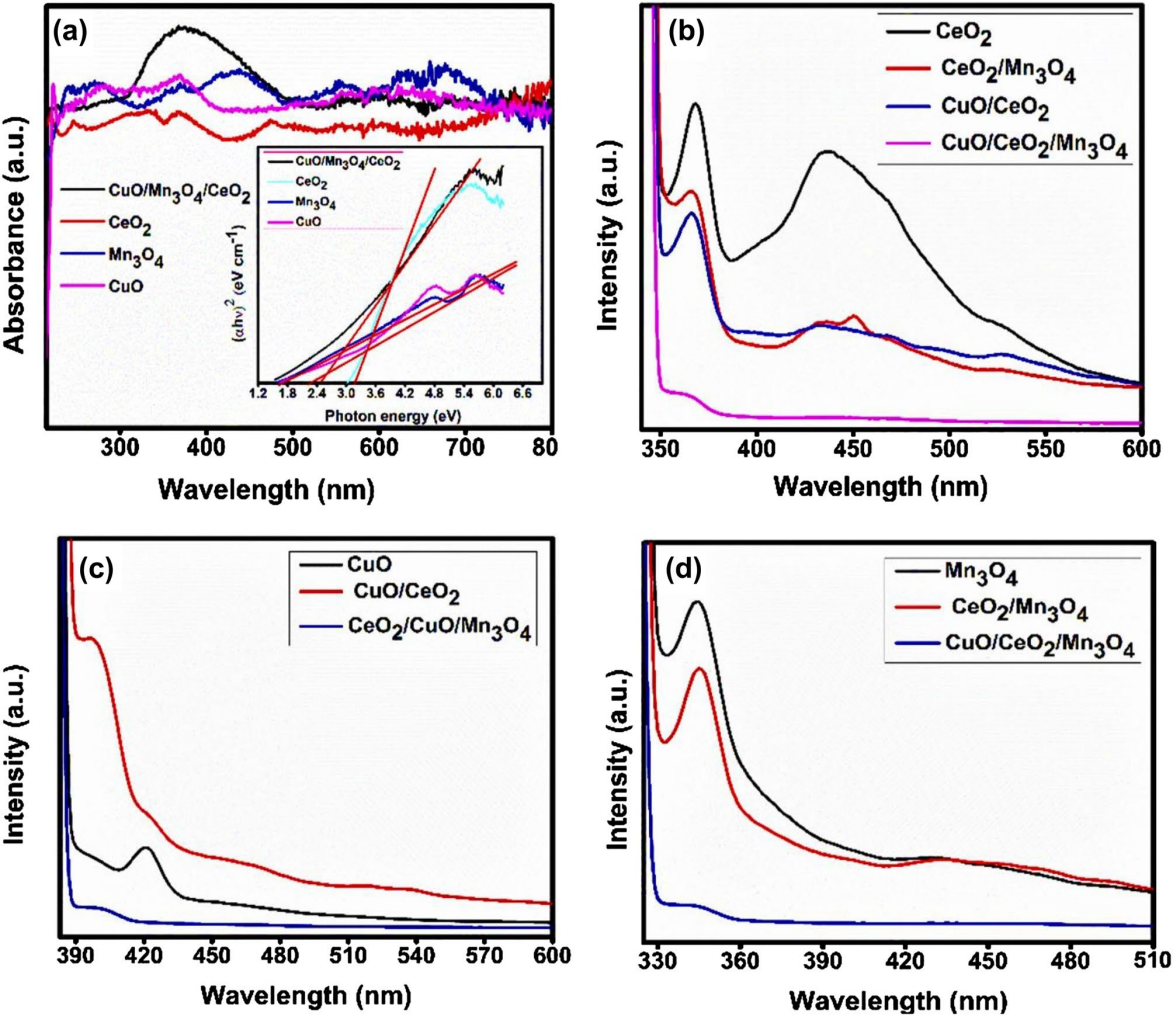
(**a**) UV- absorbance spectrum with their insect Tauc's energy band gap plots of different nano-sizes samples with CuO/Mn_3_O_4_/CeO_2_ heterostructure at (**b**–**d**) photoluminescence plots of various samples for comparative study.

The UV–Vis absorbance spectrum (Fig. 7a) of CMCu, CeO_2_, Mn_3_O_4_, and CuO were measured so that the optical characteristics of these materials could be evaluated. It was observed that the CeO_2_ in its pristine form has the highest absorption at a wavelength of less than ~ 345 nm. The absorbance spectrum of Mn_3_O_4_ showed a hump that was centered at about ~ 435 nm, while the highest absorbance of pure CuO was recorded at ~ 367 nm. In a similar manner, CMCu reacted over the whole of the visible spectrum, with the highest absorbance occurring at a wavelength of ~ 380 nm. Further, the Tauc's plots of CMCu with pristine metal oxides were plotted and given inset of Fig. 7a to determine their corresponding direct energy band gaps and, that indicated pristine CeO_2_ nanomaterials, Mn_3_O_4_ nanomaterials, and CuO nanomaterials had energy band gaps measuring ~ 3.27 eV, ~ 2.21 eV, and ~ 1.79 eV, respectively. The band gap of the final nanohybrid CMCu was around ~ 2.44 eV. The 'red shift' in the absorbance edges of the nanocomposites, which occurred while the CMCu (ternary photocatalyst) was experiencing the highest shift, provided additional confirmation of the creation of integrated photocatalysts with strong interfacial contacts. A plausible orbital intermixing in the valence shells of Ce, Cu, and Mn species could also be predicted, resulting in the formation of a conduction band (CB) at a lower magnitude of energy in the nanocomposites which was shown in the experiment^49^.

The principal cause of Photoluminescence (PL) spectra in a semiconductor material is the downward electronic transition from the conduction band (CB) to the valence band (VB), and the intensity of the PL spectra may be used as a measurement of the rate of recombination of electron–hole pairs. Therefore, the binary and final nanocomposites were evaluated alongside the unadulterated, pristine samples. The PL spectra of CeO_2_ were acquired by excitation of the material at 345 nm and at the same wavelength, CeO_2_/CuO, Mn_3_O_4_/CeO_2_, and CMCu were excited as well, and the spectra of these four systems were compared (Fig. 7b). While emissions that occurred at ~ 450 nm may be related to oxygen defects^50^, the PL-emission signal at 370 nm could be attributable to 'exciton–exciton collision'^51^. These PL emissions exhibited lower intensities in the binary metal oxide nanohybrids and the least intensity in the CMCu (ternary nanohybrid). Similarly, the spectra of CuO, CeO_2_/CuO, and CMCu were obtained after being stimulated at a wavelength of 367 nm (Fig. 7c). The very first emission peak in the CuO PL-emission spectra, located at approximately 397 nm, might be attributed to the 'radiative exciton annihilation,' and some other light humps, located at higher wavelengths, could be the result of defect levels in the metal oxides^52^. Again, it was observed that emission intensities of nanohybrids were significantly decreased, whereas the ternary nanocomposite CMCu had shown the least emission intensity. In order to make a comparison with Mn_3_O_4_, Mn_3_O_4_/CeO_2_ and CMCu were stimulated at 435 nm (Fig. 7d)^53^. Radiative recombination was at its strongest in the pristine sample, then decreased in the binary nanocomposites, and finally reached its lowest point in the CMCu nanocomposite. According to all of these findings, it seems that the final ternary nanocomposites may be capable of achieving a significant separation between the photogenerated pairs of electrons and holes.

#### XPS

**Figure 8:**
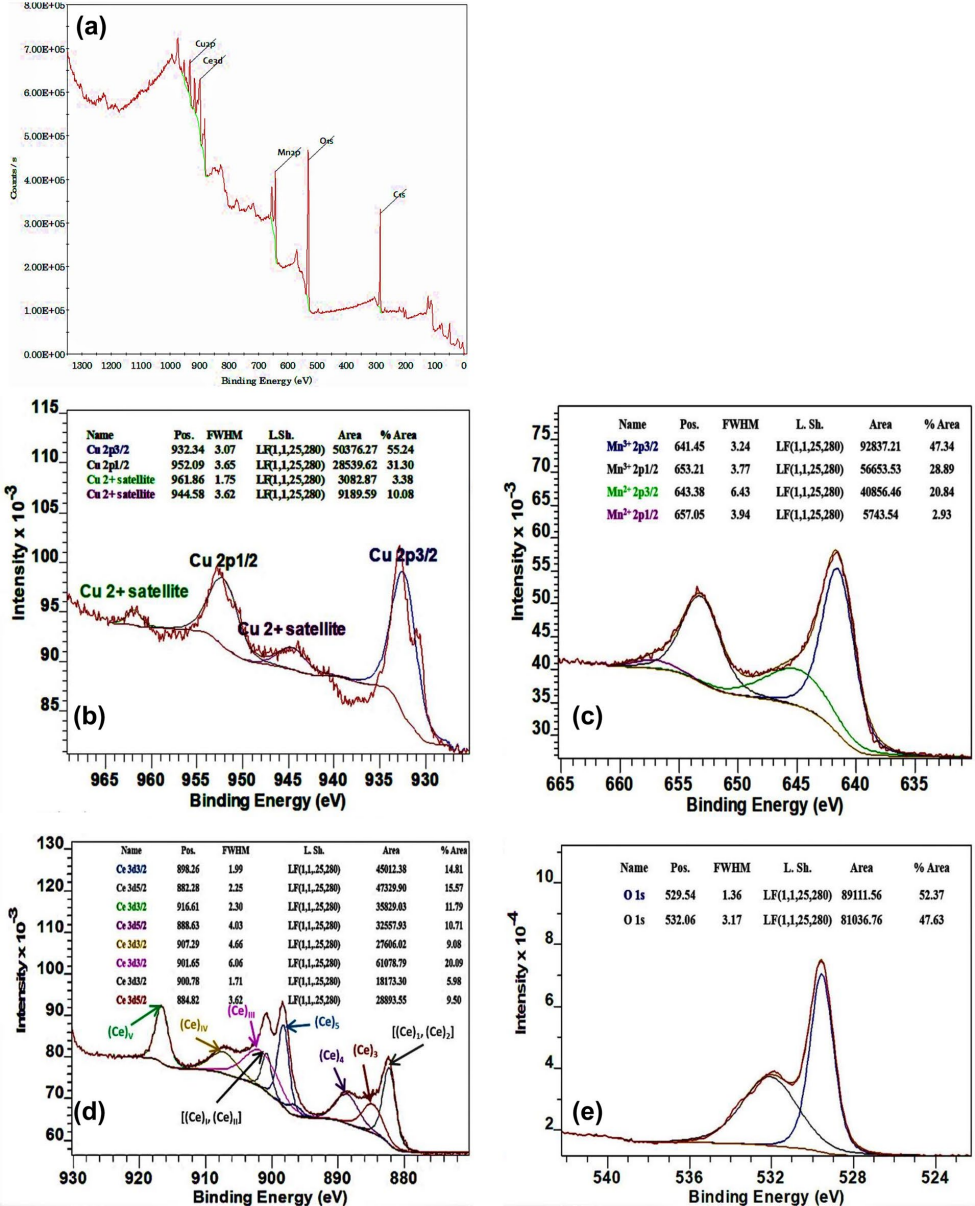
(**a**) XPS survey spectrum of CuO/Mn_3_O_4_/CeO_2_ heterostructure, XPS spectrum of (**b**) Cu2p, (**c**) Mn2p, (**d**) Ce3d, and (**e**) O1s for electronic state investigation in CuO/Mn_3_O_4_/CeO_2_ heterostructure.

The X-ray photoelectron spectroscopy (XPS) survey spectra of CuO/Mn_3_O_4_/CeO_2_ (Fig. 8a) exhibited signals that may be attributed to Cu, Mn, Ce, and O. The peaks identified at 932.34 eV and 952.09 eV in the XPS spectra of Cu 2p as shown in Fig. 8b, matched to the Cu 2p3/2 and Cu 2p1/2 with the existence of two prominent satellite signals at 944.86 eV and 944.58 eV verified the existence of Cu^+2^ in the CMCu^54,55^. The spectra of Mn 2p reveal the peaks owing to 'spin–orbit splitting' at 641.45 eV and 653.21 eV, which resemble Mn 2p3/2 and Mn 2p1/2, respectively, as shown in Fig. 8c ^56^. The difference in energy between them of 11.76 eV further validated the creation of the Mn_3_O_4_ phase in the nanocomposite. Both Mn 2p3/2 and Mn 2p1/2 signals were deconvoluted into four different signals at ~ 641.45 eV, 653.21 eV, 643.38 eV, and 657.05 eV corresponding to Mn (III) 2p3/2, Mn (III) 2p1/2, Mn (II) 2p3/2, and Mn (II) 2p1/2^57^. The bands designated Ce_1_, Ce_2_, Ce_3_, Ce_4_, and Ce_5_ with Ce_I_, Ce_II_, Ce_III_, and Ce_IV_ in the high-resolution XPS spectra of Ce3d indicate satellite characteristics emerging from corresponding Ce3d5/2 and Ce3d3/2 states^58^. The major spikes at Ce_3_ (884.8 eV), Ce_4_ (888.6 eV), Ce_5_ (898.2 eV), and Ce_III_ (901.6 eV), Ce_1V_ (907.2 eV) are also indicative of Ce^4+^ electronic states. The detection of spikes at places denoted as Ce_1_, Ce_2_, and Ce_I_, Ce_II_ provided conclusive evidence for the existence of Ce^3+^ electronic states. As a result, the Ce3d spectra are made up of a combination of Ce ions with the charge states + 3 and + 4 (Fig. 8d). The XPS spectra of O1s reveal a signal at 529.5 eV that may be attributed to the Ce–O bond in the CeO_2_ moiety^59^. The peak at 532.06 eV is most likely owing to different oxygen vacancies and surface-adsorbed oxygen species (Fig. 8e)^60,61^. The modest variations seen in the values of metal oxide binding energies (BE) compared to those published in earlier research, revealed the 'interfacial interactions' between the different moieties (metal oxides), so confirming that the planned linked photocatalyst system was successfully fabricated.

### Optimization of different operating parameters for photodegradation of MG dye using CMCu photocatalyst

#### Photocatalyst dose optimization

**Figure 9:**
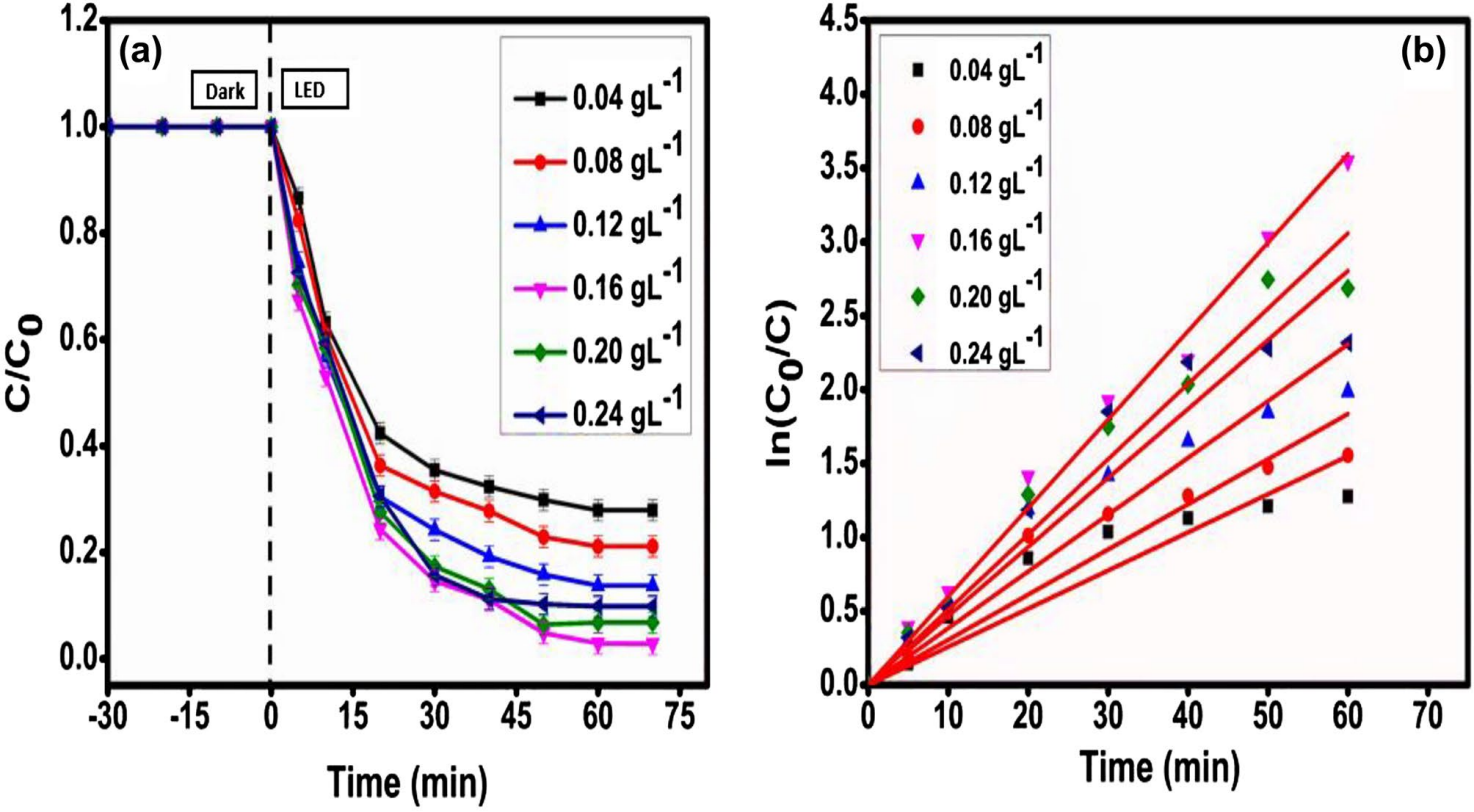
(**a**) Degradation profile and (**b**) kinetics plot for CMCu photocatalyst dose optimization.

In order to prevent the unnecessary application of the developed photocatalyst CMCu, it was necessary to conduct the screening to evaluate its ideal loading amount. Consequently, the developed photocatalyst could be used to its full potential and achieve the highest possible efficiency level. Figure 9a displays the rate of Malachite green photodegradation that occurs in the presence of varying dosages of the nanocomposite CMCu, at an initial pH of 7, and a 50 mL solution containing 50 mgL^−1^ of Malachite green was used. The catalyst intake was varied in the range of 0.04–0.24 gL^−1^ so that the optimal quantity of the developed photocatalyst necessary for Malachite green degradation could be determined. A dose concentration of 0.16 gL^−1^ of the CMCu yielded the highest photodegradation efficiency of malachite green, and beyond this, there was a little drop in efficiency. Even though further deployments of the photocatalyst would mean adding even more 'active sites' to its surface, there is significant 'solution opacity' that causes the photocatalytic efficiency to decrease. At 0.16 gL^−1^ of photocatalyst dose for 50 mgL^−1^ of Malachite green dye concentration, the velocity constant achieved its highest value of 0.0599 min^−1^ (with R^2^ = 0.99603) (Fig. 9b and Table 2), and with the photodegradation efficiency of 97.12%.

**Table 2:** Evaluation of various parameters for catalyst dose optimization for MG dye degradation.

CMCu dose (gL^−1^)	Degradation (%)	k (min^−1^)	R^2^
0.04	72.083	0.02584	0.94347
0.08	78.877	0.03059	0.95627
0.12	86.263	0.03845	0.96606
0.16	97.12	0.0599	0.99603
0.20	93.18	0.05097	0.98529
0.24	90.135	0.04673	0.96496

### Optimization of MG dye concentration

**Figure 10:**
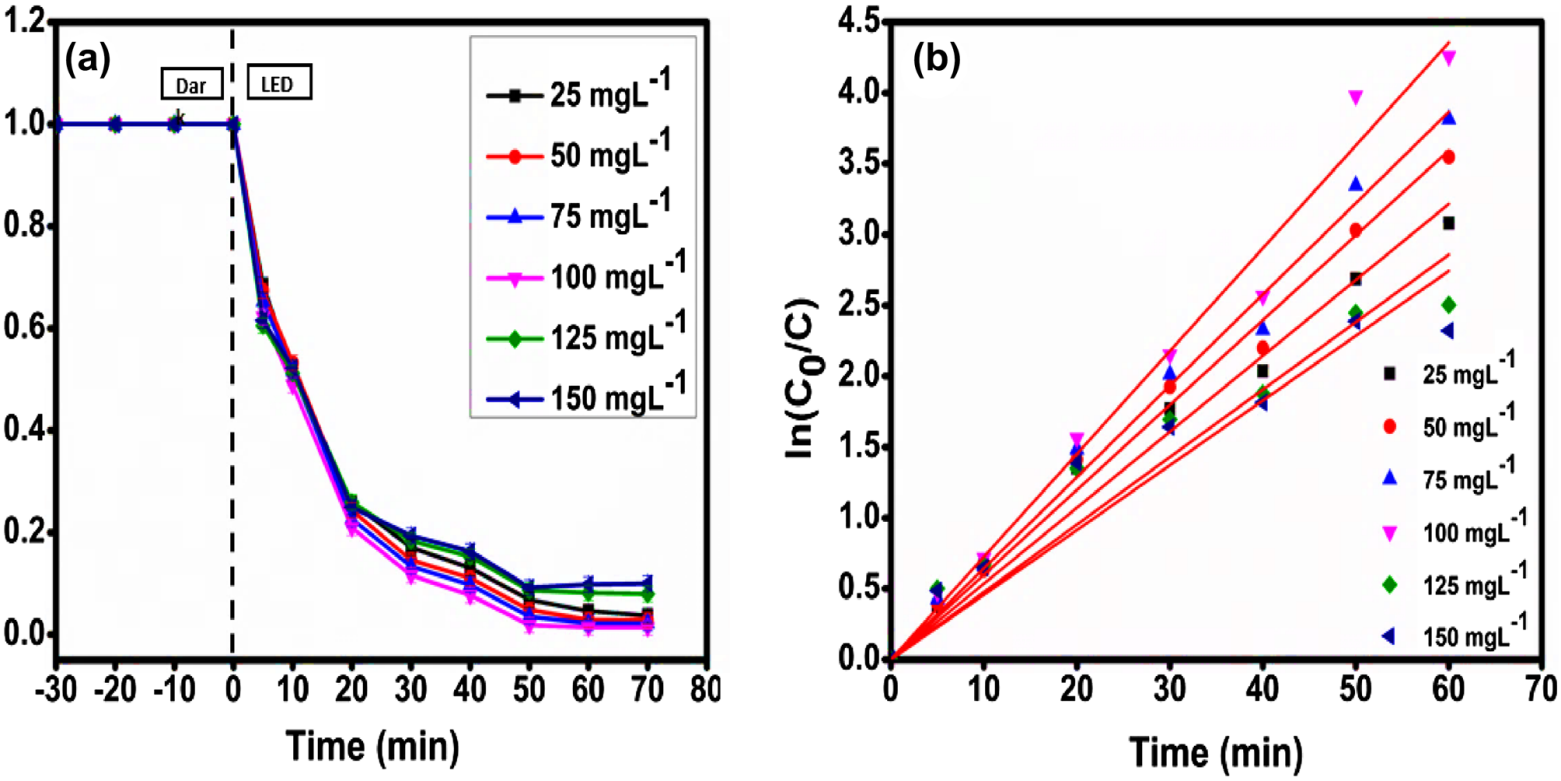
(**a**) Degradation profile and (**b**) kinetics plot for MG dye concentration optimization.

Studies were conducted using the optimum dose of the catalyst, i.e., 0.16 gL^−1^, and at a pH of 7 with varying concentrations of Malachite green dye 25–125 mgL^−1^ to evaluate how the initial concentration of the dye impacts the photodegradation activity of the catalyst CMCu. At 100 mgL^−1^ of Malachite green dye concentration, maximum efficiency of MG dye degradation was observed (Fig. 10a), which was 98.06%. A modest drop in the efficiency of the MG dye degradation was noticed at dye concentrations higher than its optimum value. Higher MG dye concentrations may be responsible for the decrease in photon path length^62^. Moreover, higher concentrations of MG dye would demand more surface area of CMCu photocatalyst for further photodegradation, which could only be accomplished by adding an extra amount of CMCu, which would inevitably increase the opacity of the solution^63^. In order to get the highest yield of photodegradation, the MG dye dosage was optimized. At the optimum concentration of the MG dye, the magnitude of the velocity constant was recorded, which was 0.07259 min^−1^ with R^2^ = 0.99357 (Fig. 10b and Table 3).

**Table 3:** Evaluation of various parameters for optimization for MG dye degradation.

MG dye conc. (mgL^−1^)	Degradation (%)	k (min^−1^)	R^2^
25	96.29	0.0536	0.99389
50	97.12	0.0599	0.99603
75	97.68	0.06444	0.9959
100	98.06	0.07259	0.99357
125	92.07	0.04764	0.97489
150	90.021	0.04572	0.96573

### Optimization of the pH of the solution

**Figure 11:**
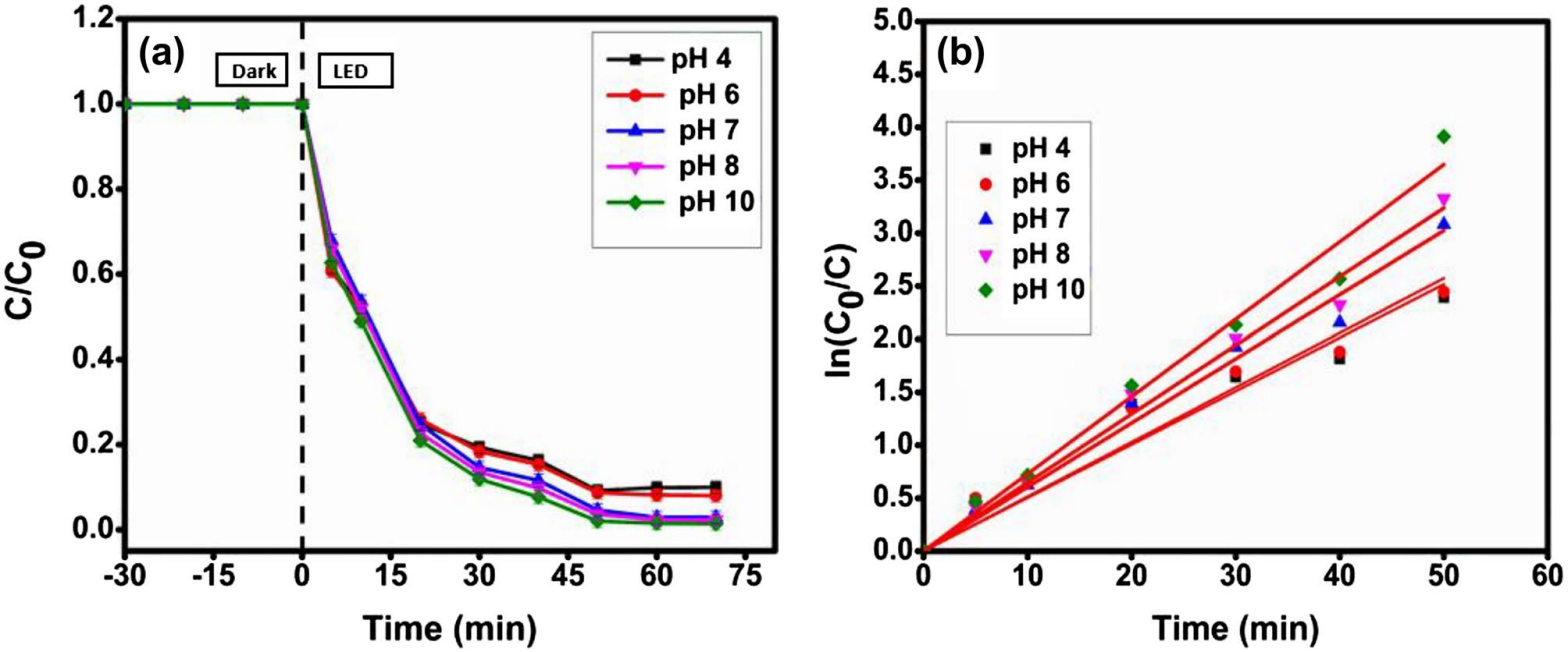
(**a**) Degradation profile and (**b**) kinetics plot for MG dye degradation at various pH environments.

The influence of pH on the photodegradation of the aqueous MG dye solution using a CMCu photocatalyst was examined. In this regard, the dose of the photocatalyst was kept constant at 0.16 gL^−1^, and the MG dye concentration was kept constant at 100 mgL^−1^ throughout the studies. On the other side, the initial pH of the dye solution was adjusted within the range of 4 to 10 by adding the corresponding amount of concentrated HCl and NaOH solutions. A slight rise in photodegradation efficiency was observed up to pH > 7, while for pH < 7, a slight drop was observed (Fig. 11a). It was determined using the mass titration method that the pH_ZPC_ (point of zero charges) of the photocatalyst is approx. 6.4. Therefore, a high concentration of hydroxide ions could be generated on the photocatalyst surface when the solution pH rises beyond the value of pH_ZPC_^64^. In an environment with a higher pH, the accumulation of hydroxide ions on the surface of the photocatalyst could cause the catalyst to attract the electron-deficient cationic MG dye molecules, while at pH lower than pH_ZPC,_ the photodegradation of MG dye declined because of the accumulation of H^+^ ion concentration over the catalyst surface which repels the cationic MG dye molecules^1^. As can be observed from pseudo-first-order kinetics (Fig. 11b), the velocity constant reached its highest value at a pH of 10, which is 0.07295 min^−1^ (Table 4).

**Table 4:** Evaluation of various parameters for optimization of pH of the photocatalysis environment for MG dye degradation.

pH	Degradation (%)	k (min^−1^)	R^2^
4	90.03	0.05034	0.97605
6	92.04	0.05147	0.97991
7	98.06	0.06049	0.99275
8	98.64	0.06475	0.99365
10	98.98	0.07295	0.99144

### Optimization of LED exposure time

**Figure 12:**
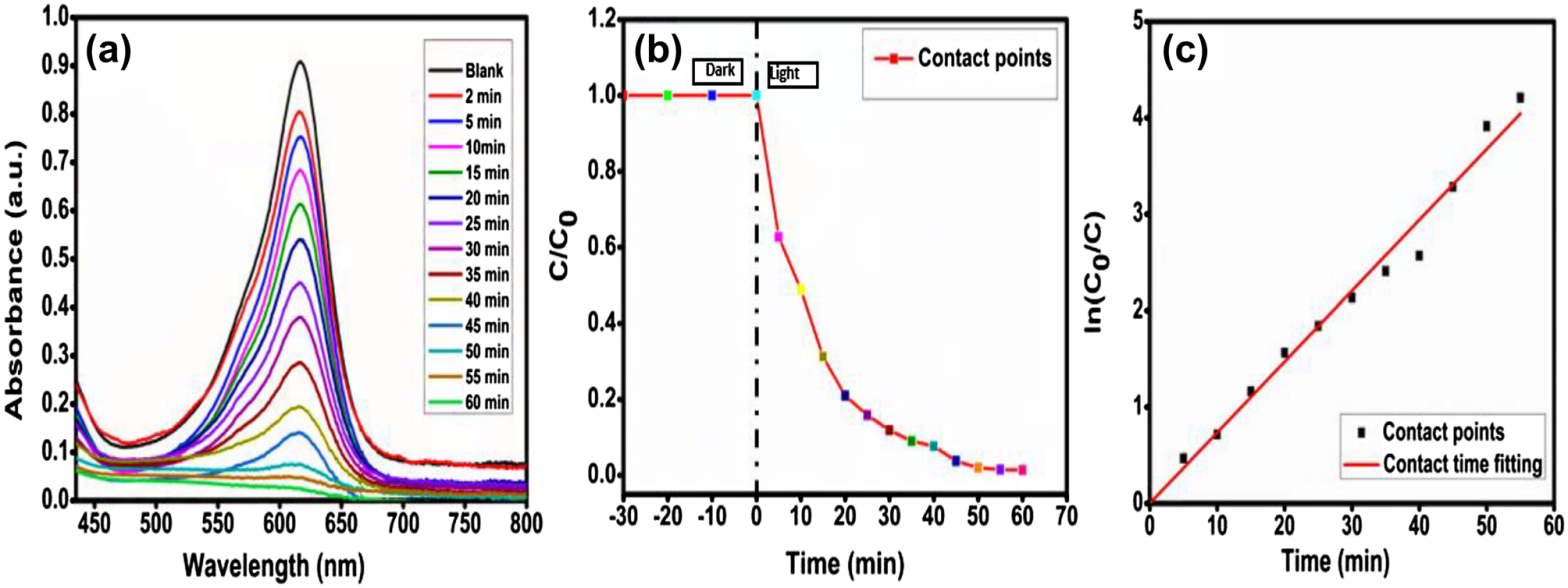
(**a**) Contact time degradation profile, (**b**) Degradation profile, and (**c**) kinetics plot for MG dye degradation at a different time interval.

By monitoring the degradation output over time at various intervals while performing under the various optimal parameters of photocatalyst dose, initial concentration of dye, and pH, the influence of LED exposure time was examined. The photocatalyst CMCu loading utilized in this study was 0.16 gL^−1^, with an initial MG dye concentration of 100 mgL^−1^ and a pH > 7. After 60 min, the maximum MG dye photodegradation of 98.98% was recorded (Fig. 12a, b). The kinetic for the contact time is also provided in Fig. 12c, which results in the velocity rate constant value of 0.07352 min^−1^ with R^2^ = 0.9956. After 60 min, the light-driven photodegradation reaction was stopped because of photocatalyst's active sites had been nearly exhausted.

### Study for reusability and durability of the photocatalyst

**Figure 13:**
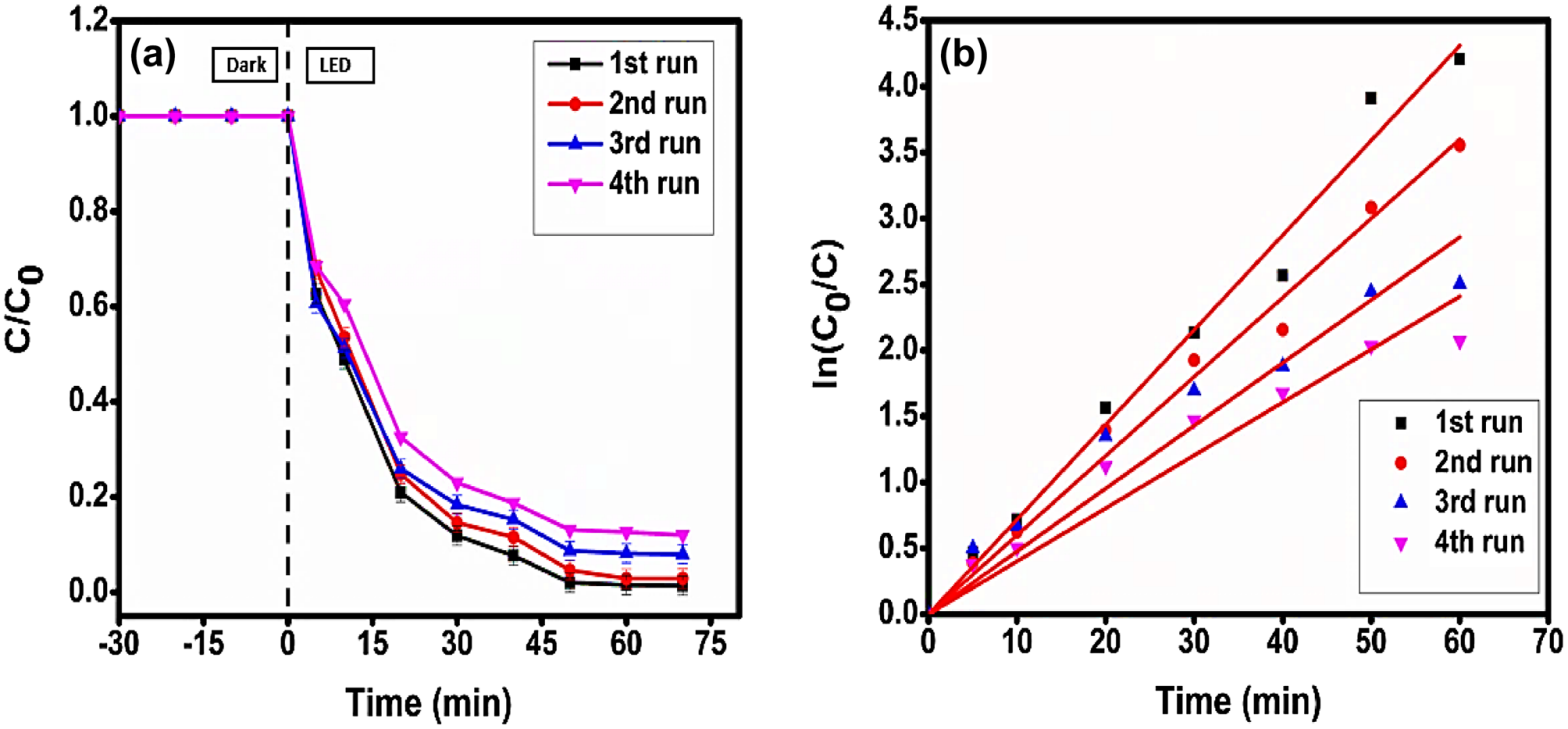
(**a**) Degradation profile and (**b**) kinetics plot up to four cycles of reused photocatalyst for MG dye degradation.

**Figure 14:**
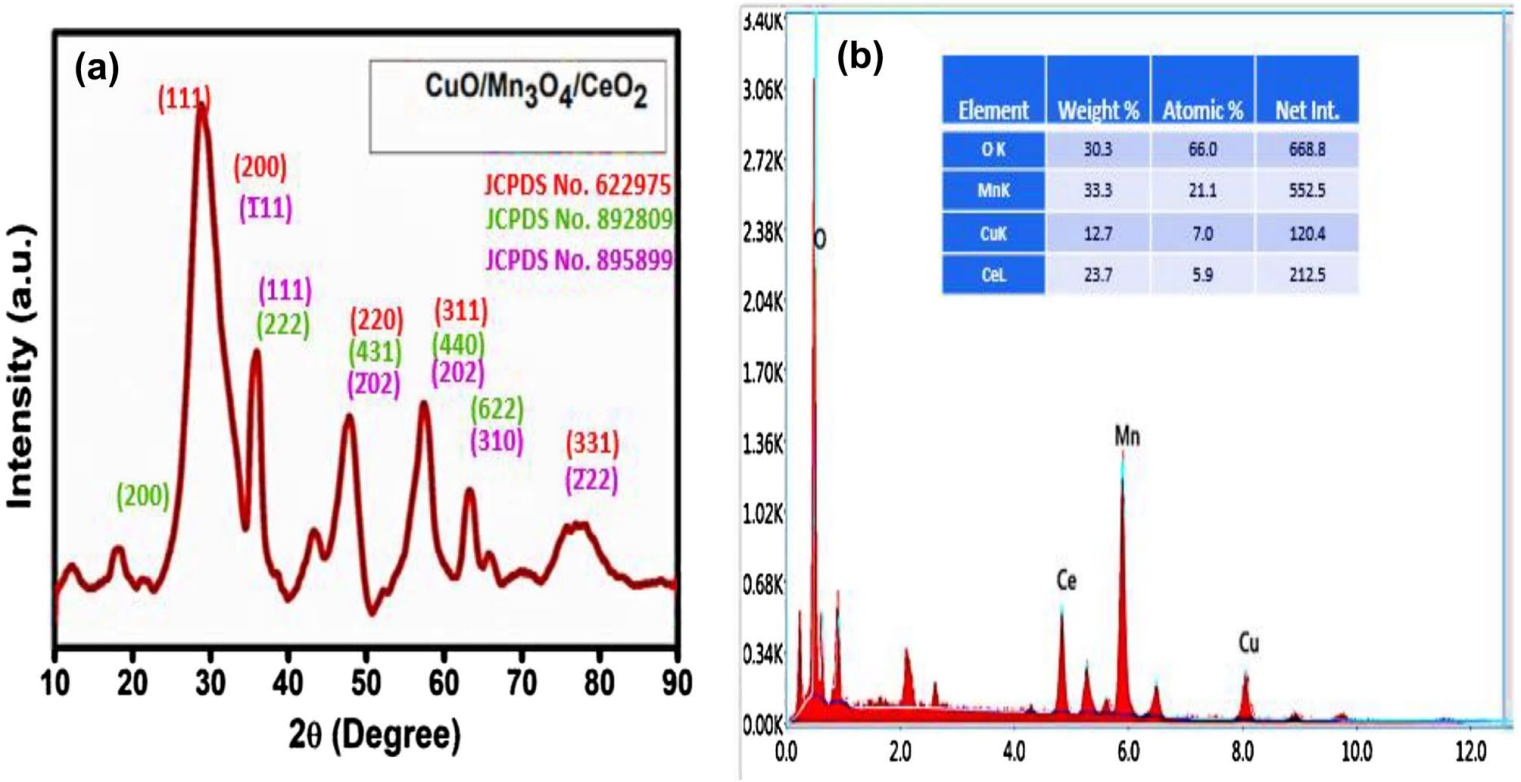
(**a**) PXRD and (**b**) EDAX spectrum of photocatalyst CuO/Mn_3_O_4_/CeO_2_ after the run of the fourth cycle for MG dye degradation.

The heterostructure photocatalyst that had been developed was recovered, and it was found that it could be utilized up to four runs in a row (Fig. 13a, b). The recovered catalyst was separated using centrifugation at 1000 rpm. After that, it was cleaned many times with distilled water and ethanol before being dried at a temperature of 90 °C for 2 h. The processed photocatalyst was used once again in the subsequent experimental run. Both the degradation efficiency and the pseudo-first-order rate constant revealed a little drop (Table 5), which could be explained by the fact that the coupled unit of photocatalyst had been somewhat dislodged. Further, the XRD data that was produced for the regenerated photocatalyst indicated that the typical crystallographic planes that were present in CuO/Mn_3_O_4_/CeO_2_ before usage were also present in the regenerated photocatalyst material (Fig. 14a). This demonstrated that the developed photocatalyst has a high degree of overall durability. Moreover, it was further validated by the outcomes of the EDAX spectra of the regenerated photocatalyst (Fig. 14b), which revealed very little variation from the information derived for the photocatalyst before use. These observations showed that the developed ternary nano-scaled composite was stable, which indicated that there was extensive interfacial coupling among the components, and they provided great proof of low leaching of metal ions throughout the photocatalytic activity. The synthetic method that was used to develop the hierarchical nanostructure may have been a factor in achieving excellent elemental compatibility with excellent dispersion quality of the photocatalyst CMCu, as evidenced by EDAX, Transmission electron micrographs, optical data, and X-ray photoelectron spectrum, and this resulted in lowering of accumulation of any particular metal oxide within ternary photocatalyst CMCu. Additionally, some articles suggest that semiconductors with a hierarchical system of nanostructure have more excellent resistance to accumulation^65^. As a result, the hierarchical design will make it more difficult for metal ions to leach out of aggregates, which is caused by accumulation.

**Table 5:** Evaluation of various parameters for reusability test of photocatalyst for MG dye degradation.

Run	Degradation (%)	k (min^−1^)	R^2^
1st	98.98	0.07295	0.99433
2nd	96.12	0.06126	0.9955
3rd	92.48	0.04764	0.97508
4th	88.72	0.04015	0.97469

### Comparative study of different catalysts for degradation of Malachite green dye

**Figure 15:**
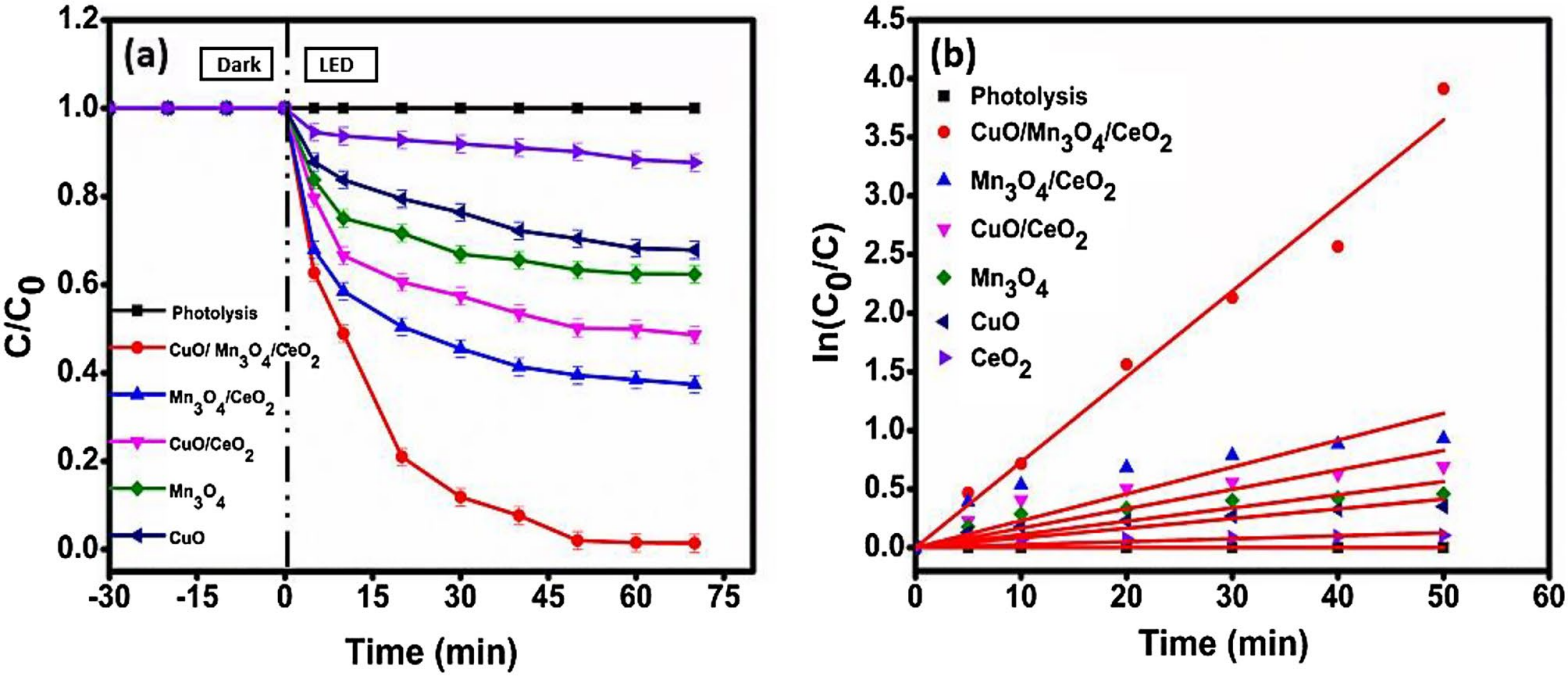
(**a**) Degradation profile and (**b**) kinetics plot for comparative study of different samples of the catalyst with photocatalyst CMCu for MG dye degradation.

Photolysis, along with the pristine CeO_2_ nanomaterial, scarcely accomplished any appreciable photocatalytic activity (Fig. 15a, b). CuO/Mn_3_O_4_/CeO_2_ displayed a photocatalytic degradation efficiency of 98.98 ± 1.5% within 60 min, whereas Mn_3_O_4_/CeO_2_ and CuO/CeO_2,_ Mn_3_O_4,_ and CuO could achieve photocatalytic degradation efficiencies of 62.61%, 51.42%, 37.64%, and 32.16% under the equal time duration and identical set of circumstances (Fig. 15a, b). During photocatalytic degradation over CuO/Mn_3_O_4_/CeO_2_, an exceptional rate constant of 0.07295 min^−1^ was recorded. In terms of the percentage of photocatalytic degradation, the ternary nanocomposite performed 1.58-fold higher than the Mn_3_O_4_/CeO_2_ combination and 1.92-fold higher than the CuO/CeO_2_ combination. Additionally, the ternary photocatalyst was shown to be astonishingly more effective than CuO-Gd_2_Ti_2_O_7_, which required 90 min to produce 88.60% of Malachite green dye degradation at a rate constant of 0.0198 min^−1^^66^ and SnO_2_/ZnO flower like composite which requires 150 min for 98% of Malachite green dye degradation with the velocity constant of 0.00168 min^−1^^67^. Table 6 presents the results of a comparison between the photodegradation performance of the newly synthesized photocatalyst and that of the catalysts that have been reported in the past.

**Table 6:** comparative study of various samples of catalyst and previously reported catalyst with designed photocatalyst CuO/Mn_3_O_4_/CeO_2._

Catalyst	Degradation efficiency (%)	Light exposure time (min)	k (min^−1^)	R^2^
CuO/Mn_3_O_4_/CeO_2_	98.98	60	0.07295	0.99144
CuO-Gd_2_Ti_2_O_7_^66^	88.60	90	0.0198	0.89128
SnO_2_/ZnO^67^	98	150	0.00168	0.99412
Mn_3_O_4_/CeO_2_	62.61	60	0.02289	0.89797
CuO/CeO_2_	51.42	60	0.01655	0.90646
Mn_3_O_4_	37.64	60	0.01126	0.89
CuO	32.16	60	0.00827	0.92638
CeO_2_	12.34	60	0.00252	0.86249

### Trapping agents (scavengers) experiment

**Figure 16:**
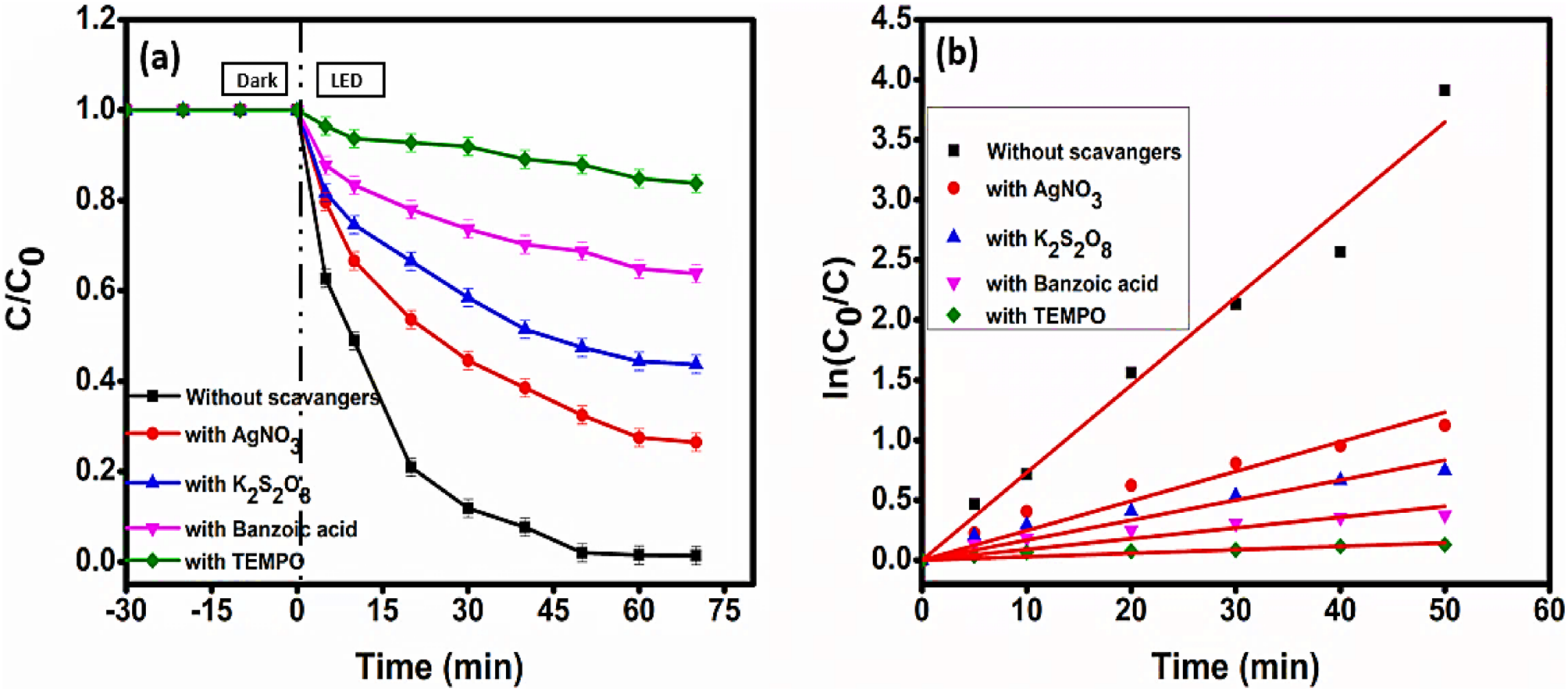
(**a**) Degradation profile and (**b**) kinetics plot for scavenger experiment for MG dye degradation with photocatalyst CMCu.

Photocatalytic degradation studies were carried out with quenchers to evaluate the important characteristics performed by various reactive species, such as ${\mathrm{O}}_{2}^{2-}$, ·OH, e − , and h^+^ towards the photodegradation of MG dye. For scavenging the ${\mathrm{O}}_{2}^{2-}$, ·OH, e − , and h^+^, the corresponding scavengers (trapping agents), TEMPOL (4-hydroxy-2,2, 6,6- tetramethyl piperidinyl oxy), C_7_H_6_O_2_ (Benzoic acid), K_2_S_2_O_8_ (Potassium persulfate)_,_ and AgNO_3_ (silver nitrate), respectively, were used. The photodegradation experienced an apparent retardation in the vicinity of TEMPOL^68^ and BA^69^ (Fig. 16a, b), and it was accompanied by a decline in efficiencies, which went from 98.98% with CuO/Mn_3_O_4_/CeO_2_ photocatalyst to 16.42% and 34.28%, respectively. Interestingly, the drop in photodegradation efficiencies was not as significant when K_2_S_2_O_8_^70^ and AgNO_3_^71^ were present. Based on the information mentioned above, substantial roles in the photodegradation of Malachite green dye are likely played by reactive oxygen species (ROS), ${\mathrm{O}}_{2}^{2-}$ and ·OH radicals^72,73^.

### Plausible mechanistic approach for photodegradation of Malachite green dye by ternary nanocomposite CuO/CeO_2_/Mn_3_O_4_

**Figure 17:**
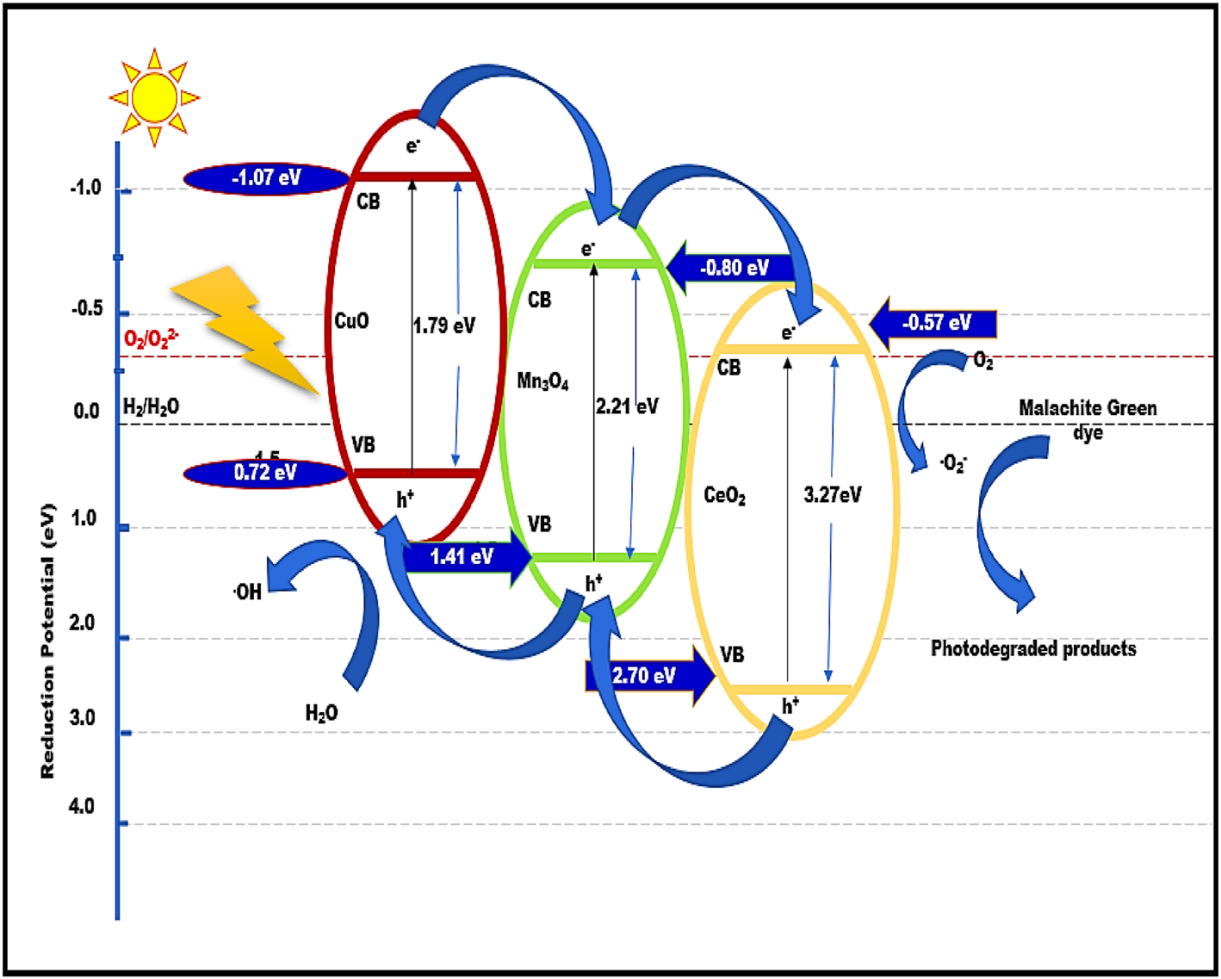
(**a**) Schematic presentation of plausible mechanism for photodegradation of Malachite green dye using designed heterostructure CuO /Mn_3_O_4_/ CeO_2._

The spectacular photodegradation efficiency of the fabricated nano-heterostructure as a photocatalyst provided an efficient demonstration of the photocatalytic activity of the integrated photocatalyst. Furthermore, PL results clearly demonstrated the segregation of photo-induced electron–hole pairs. Moreover, the nano-sized photocatalyst exhibited a level of efficiency that was quite constant up to the fourth cycle. The above observations are consequently suggestive of the establishment of strong interfacial reactions among the participating components. The plausible mechanism of CuO/Mn_3_O_4_/CeO_2_ photocatalysis is shown in Fig. 17. With reference to a normal hydrogen electrode (NHE), the potential edge value of the CB (conduction band) for CuO is located at − 1.07 eV^74^, while for Mn_3_O_4_, it is located at − 0.80 eV^75^, and CeO_2_ has a CB edge potential at − 0.57 eV^76^. CB of CuO will always have a greater negative edge potential value than the CB edge potential value of Mn_3_O_4_, which will, in response, have a greater negative CB edge potential value than CeO_2_. Consequently, as a response to the photon absorption from the source of visible light, electrons (e^−^) are moved from the VB (valence band) of CuO towards its own CB These electrons are then moved towards the conduction band of semiconductor Mn_3_O_4_, and subsequently, they concentrate at the Conduction band of nanomaterial CeO_2_. At the same time, holes (h^+^) follow the opposite direction movement from the Valence band of CeO_2_ to the Valence band of semiconductor Mn_3_O_4_, with a further move towards the valence band of semiconductor CuO. Afterward, the electrons concentrated at the Conduction band of CeO_2_ interacted with the oxygen molecules (O_2_) that had been adsorbed over the surface of the reaction system, which resulted in the generation of superoxide anion radicals (^.^ O_2_^−^). This is due to the fact that the potential edge value of CB of CeO_2_ is lower than the typical reduction potential of O_2_/^.^O_2_^-^, which is − 0.33 eV^77^. The holes, on the other side, generated hydroxyl radicals (·OH) by reacting with water molecules (H_2_O). It's possible that the pathways indicated below will lead to the formation of ·OH radicals. Equations 4–13 below provide the best description of the synergistic effect that exists among the various components that make up the integrated photocatalyst, which ultimately results in the superior photocatalytic activities of the integrated photocatalyst^22^^,^^78–80^

4 $$\mathrm{CuO}+\mathrm{hv}\left(\mathrm{visible light}\right)={\mathrm{CuO}}_{\left({\mathrm{e}}_{\mathrm{CB}.}^{-}\right)}+{\mathrm{CuO}}_{\left({\mathrm{h}}_{\mathrm{VB}.}^{+}\right)}$$

5 $${\mathrm{CuO}}_{\left({\mathrm{e}}_{\mathrm{CB}.}^{-}\right)}+{\mathrm{Mn}}_{3}{\mathrm{O}}_{4}=\mathrm{CuO}+{{\mathrm{Mn}}_{3}{\mathrm{O}}_{4}}_{\left({\mathrm{e}}_{\mathrm{CB}.}^{-}\right)}$$

6 $${{\mathrm{Mn}}_{3}{\mathrm{O}}_{4}}_{\left({\mathrm{e}}_{\mathrm{CB}.}^{-}\right)}+{\mathrm{CeO}}_{2}={\mathrm{Mn}}_{3}{\mathrm{O}}_{4}+{{\mathrm{CeO}}_{2}}_{\left({\mathrm{e}}_{\mathrm{CB}.}^{-}\right)}$$

7 $${{\mathrm{CeO}}_{2}}_{\left({\mathrm{e}}_{\mathrm{CB}.}^{-}\right)}+{\mathrm{O}}_{2}={{\mathrm{CeO}}_{2}}_{\left({\mathrm{e}}_{\mathrm{CB}.}^{-}\right)}+ {.\mathrm{O}}_{2}^{-}$$

8 $${\mathrm{h}}_{\mathrm{VB}.}^{+}+{\mathrm{H}}_{2}\mathrm{O}= .\mathrm{OH}+ {\mathrm{H}}^{+}$$

9 $${\mathrm{h}}_{\mathrm{VB}.}^{+}+{\mathrm{H}}_{2}\mathrm{O}={\mathrm{H}}_{2}{\mathrm{O}}_{2}+ {2\mathrm{H}}^{+}$$

10 $${\mathrm{H}}_{2}{\mathrm{O}}_{2}+ {\mathrm{e}}^{-}= .\mathrm{OH}+ {\mathrm{OH}}^{-}$$

11 $${.\mathrm{O}}_{2}^{-}+ {2\mathrm{H}}^{+}+ {\mathrm{e}}^{-}= {\mathrm{H}}_{2}{\mathrm{O}}_{2}$$

12 $${\mathrm{H}}_{2}{\mathrm{O}}_{2}+ {\mathrm{e}}^{-}= .\mathrm{OH}+ {\mathrm{OH}}^{-}$$

13 $${.\mathrm{O}}_{2}^{-}/.\mathrm{ O}.\mathrm{H}. +\mathrm{ Malachite\,green\,dye }\left(\mathrm{organic\,pollutant}\right)=\mathrm{ Degraded\,products}$$

### A brief investigation of the effect of various inorganic salts and different water matrices in the photodegradation of MG dye

#### Effect of inorganic ions

There is currently a dearth of knowledge on how inorganic ions affect the photodegradation efficiency of environmental pollutants. In light of the potential practical uses, this work also examined the impact of inorganic ions on the photodegradation of MG Unfortunately, because of the intricate impacts of co-existing inorganic ions, photocatalytic degradation is currently restricted in terms of practical applicability^81^. For instance, several inorganic ions, such as carbonate, chloride, fluoride, calcium, aluminum, and sodium ions, are present in water. Therefore, it is important to investigate these ions' effect on the prepared material's photocatalytic activity for its real-world practical applications as a photocatalyst.

**Figure 18:**
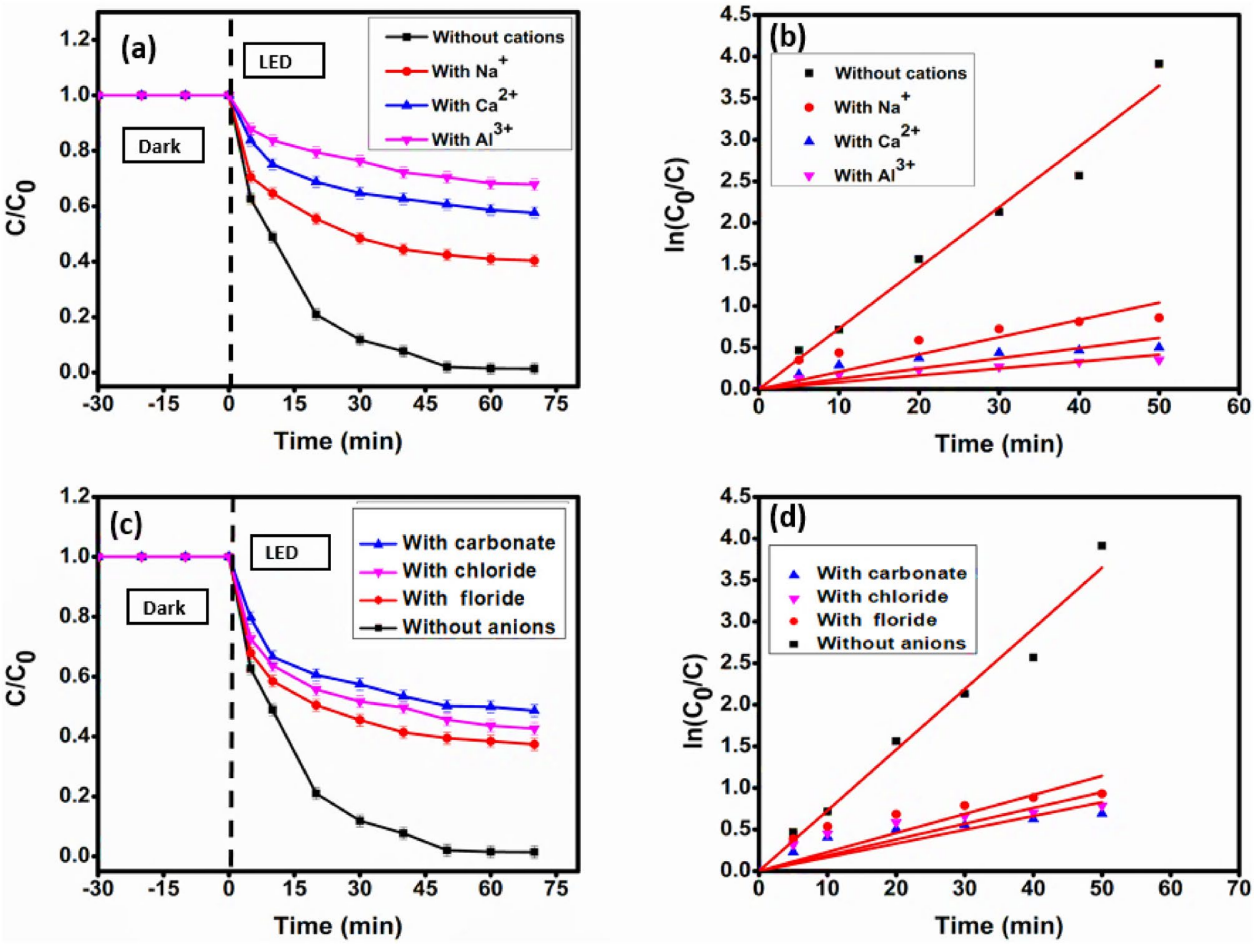
(**a**) Degradation profile and (**b**) kinetics plot in an environment of selected cations, and (**c**) Degradation profile and (**d**) kinetics plot in an environment of selected anions for MG dye degradation using photocatalyst CMCu.

To study these ions' effect, 0.1 M of Na_2_SO_4_, CaSO_4_, Al_2_(SO_4_)_3_, NaF, NaCl, and Na_2_CO_3_ are added into the reaction mixture at optimum conditions before irradiating the solution. The cations Na^+^, Ca^2+^, and Al^3+^ being in their stable oxidation states, are known to cause a negligible effect on photocatalysis^82^. As illustrated in Figs. 18a, b, the degradation of MG is inhibited to varying extents in the presence of these cations. This decrease in the photocatalytic activity might be due to the presence of sulfate anion in the salts. However, the Al^3+^ greatly inhibited the degradation of MG because of its higher charge density and the tendency to adsorb on the surface of the photocatalyst^83^. Aluminum ions blocked the active sites on the surface, thereby inhibiting the generation of reactive oxygen species (ROS). Ca^2+^ was found to slow down the degradation process a bit more than Na^+^ ions.

The wastewater also contains a number of inorganic anions, which are known to obstruct photogenerated ROS and influence the total removal of organic contaminants. As illustrated in Figs. 18c, d, the degradation efficiency is significantly hindered in the presence of anions. The negative effect of these anions on the degradation of MG follows the order CO_3_^2−^ > Cl^−^ > F^−^. This decrease could be due to two main reasons, firstly, due to the blockage of active sites of the photocatalyst inhibiting the generation of ROS, and secondly, due to the quenching effect of these anions.

It is evident from Figs. 18a, b the chloride ions g inhibited the degradation of MG because of their strong affinity towards the holes and hydroxyl radicals^84^. This quenching effect of Cl^−^ was the main reason for the suppression of the degradation efficiency, as illustrated below (Eq. 14–16) ^85^:

14 $${\text{Cl}}^{ - } + {\text{h}}^{ + } \to {\text{Cl}}^{ \cdot }$$

15 $${\text{Cl}}^{ - } + {\text{OH}}.^{ \cdot } \to {\text{Cl}}^{ \cdot } + {\text{OH}}^{ - }$$

16 $${\text{Cl}}^{ \cdot } + {\text{Cl}}^{ - } \to \cdot {\text{Cl}}_{2}^{ - }$$

After being adsorbed on the surface of the photocatalyst, the chloride ions interact with the holes and hydroxyl radicals, forming the ${\text{Cl}}^{ \cdot }$ free radicals, which further get converted to Cl^−^ ions upon the reaction with the electrons ^86^. Furthermore, chloride ions are resistant to oxidation, rendering them good inhibitors of the photodegradation reaction.

The carbonate ions are greatly known for their quenching effect of hydroxyl radicals according to the following Eq. 17^87^ following second-order-kinetics:

17 $${\text{CO}}_{3}^{2 - } + {\text{OH}}^{ \cdot } \to {\text{OH}}^{ - } + \cdot {\text{CO}}_{3}^{ - }$$

The carbonate free radicals thus formed could also bring about the degradation of MG However, due to their low oxidation potential compared to hydroxyl radicals, they only hindered the photodegradation process.

The F^−^ ions are highly stable, and due to their non-oxidizable nature and small size, they cover the surface of the photocatalyst, inhibiting the generation of hydroxyl radicals ^88,89^. Therefore, it could be confirmed that hydroxyl radicals are important species for the degradation of organic pollutants in the presence of F^−^ ions ^84^.

### Effect of water matrices

**Figure 19:**
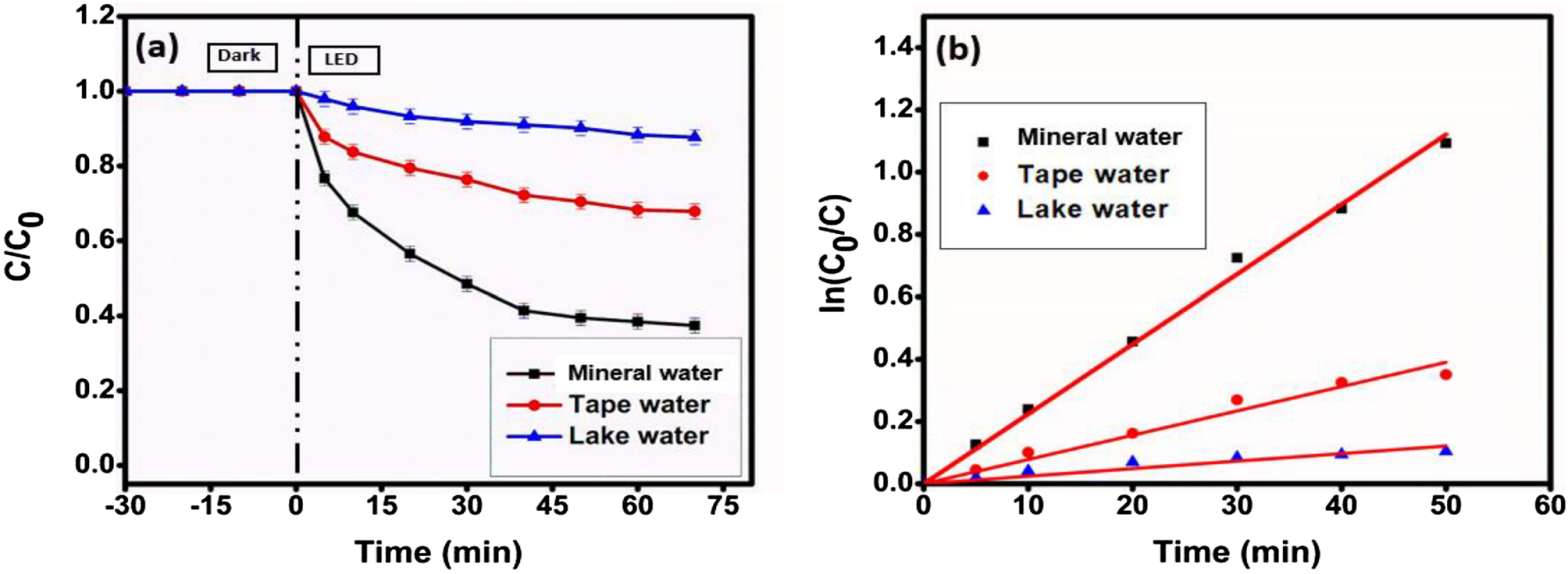
(**a**) Degradation profile and (**b**) kinetics plot for photodegradation of prepared MG dye solution in different water samples employing photocatalyst CMCu.

To further authenticate the photodegradation process for real wastewater treatment, the degradation of MG was also investigated in three different water samples. Mineral water, lake water, and tap water have a pH of ~ 7.21, ~ 8.36, and ~ 7.86, respectively, and are employed to investigate the effect of water matrices on the degradation of malachite green. The degradation profiles and kinetics for MG dye photodegradation in three different water samples are shown in Fig. 19a, b. All the water samples showed a significant retarding effect on the degradation of MG dye. Mineral water, the cleanest among all, expectedly showed a considerable degradation efficiency of 62.36%, which is better than the other two samples. Lake water contains various other co-existing ions and organic compounds that showed minimum efficiency with only 14.28% degradation of MG, while 36.12% degradation could be achieved with tap water. Light refraction and the presence of different photocatalysis-inhibiting inorganic ions and organic molecules to variable degrees in the aforementioned water matrices are two main causes of the general decrease in the photodegradation activity of the photocatalyst in these aquatic environments. The fact that the photocatalyst performed poorly in the former matrix may be due to tap water's higher mineral content than lake water.

## Conclusion

In this study, a ternary nano-heterostructure CuO/Mn_3_O_4_/CeO_2_ as a photocatalyst was designed employing simple hydrothermal methods, and the photocatalytic activity of the developed nano-heterostructure was thoroughly investigated. For determining the structural, morphological, composition, and optical characteristics, numerous characterization and analytical methods were used. Images from TEM clearly demonstrated the growth of CeO_2_ nanoparticles with cubic lattice structure in the composite. HRTEM images provide further insight into the successful fabrication of the desired ternary heterojunction and the coupling of CuO and Mn_3_O_4_ nanoparticles with CeO_2_ nanoparticles. The CMCu heterostructure has a band gap of 2.44 eV, which was evaluated according to Tauc's plot using UV-DRS data. The robust formation of a staggered type II heterojunction with a significant gap between light-induced electron–hole pairs was further supported by PL investigations. The nanostructure was shown to be photo-catalytically active in the UV–visible region of the spectrum of light, and it was capable of achieving a 98.98% photodegradation of Malachite green dye within 60 min with excellent pseudo-first-order rate constant value of 0.07295 min^−1^. Additionally, the recovered photocatalyst was repeatedly utilized and showed ~ 88.72% of photodegradation of Malachite green dye up to the fourth run. Scavengers' experiments, inorganic salt analysis, and experiments in different water matrices are briefly discussed to understand the species which influence the reaction by assisting or interfering in the reaction. Finally, a plausible mechanistic explanation is discussed to explain the path involved in the photodegradation of MG dye using CMCu photocatalyst.

## Data Availability

The authors declare that all data supporting the findings of this study are available within the article.
